# Structural properties of the HNF-1A transactivation domain

**DOI:** 10.3389/fmolb.2023.1249939

**Published:** 2023-10-16

**Authors:** Laura Kind, Mark Driver, Arne Raasakka, Patrick R. Onck, Pål Rasmus Njølstad, Thomas Arnesen, Petri Kursula

**Affiliations:** ^1^ Department of Biomedicine, University of Bergen, Bergen, Norway; ^2^ Zernike Institute for Advanced Materials, University of Groningen, Groningen, Netherlands; ^3^ Mohn Center for Diabetes Precision Medicine, Department of Clinical Science, University of Bergen, Bergen, Norway; ^4^ Section of Endocrinology and Metabolism, Children and Youth Clinic, Haukeland University Hospital, Bergen, Norway; ^5^ Department of Surgery, Haukeland University Hospital, Bergen, Norway; ^6^ Faculty of Biochemistry and Molecular Medicine & Biocenter Oulu, University of Oulu, Oulu, Finland

**Keywords:** β-cell, diabetes, transcription factor, HNF-1A, MODY, intrinsically disordered protein, liquid-liquid phase separation, short linear motif

## Abstract

Hepatocyte nuclear factor 1α (HNF-1A) is a transcription factor with important gene regulatory roles in pancreatic β-cells. *HNF1A* gene variants are associated with a monogenic form of diabetes (HNF1A-MODY) or an increased risk for type 2 diabetes. While several pancreatic target genes of HNF-1A have been described, a lack of knowledge regarding the structure-function relationships in HNF-1A prohibits a detailed understanding of HNF-1A-mediated gene transcription, which is important for precision medicine and improved patient care. Therefore, we aimed to characterize the understudied transactivation domain (TAD) of HNF-1A *in vitro*. We present a bioinformatic approach to dissect the TAD sequence, analyzing protein structure, sequence composition, sequence conservation, and the existence of protein interaction motifs. Moreover, we developed the first protocol for the recombinant expression and purification of the HNF-1A TAD. Small-angle X-ray scattering and synchrotron radiation circular dichroism suggested a disordered conformation for the TAD. Furthermore, we present functional data on HNF-1A undergoing liquid-liquid phase separation, which is in line with *in silico* predictions and may be of biological relevance for gene transcriptional processes in pancreatic β-cells.

## 1 Introduction

Hepatocyte nuclear factor (HNF-1A) is a transcription factor with essential gene regulatory functions in the pancreatic β-cells, which are important regulators of glucose homeostasis. The β-cell-specific signaling cascade “glucose-stimulated insulin secretion” (GSIS) is initiated by a rise in blood glucose levels after the ingestion of a meal and results in the secretion of insulin into the blood stream (Straub and Sharp, 2002), in turn stimulating peripheral tissues to internalize glucose from the blood. HNF-1A is indispensable for β-cell maintenance and function, as it transcriptionally regulates components of the GSIS cascade, as well as various transcription factors integrated into a gene regulatory network (Wang et al., 2002; Odom et al., 2004; Servitja and Ferrer, 2004; Servitja et al., 2009). Understanding HNF-1A function is potentially clinically relevant, as numerous genetic variants within the *HNF1A* gene can cause the hereditary diabetes type “maturity onset diabetes of the young” (HNF1A-MODY), be associated with HNF1A-MODY with reduced penetrance, or act as polygenic risk factors for type 2 diabetes (Flannick et al., 2016; Najmi et al., 2017; Krentz and Gloyn, 2020; Zhang et al., 2021).

**FIGURE 1:**
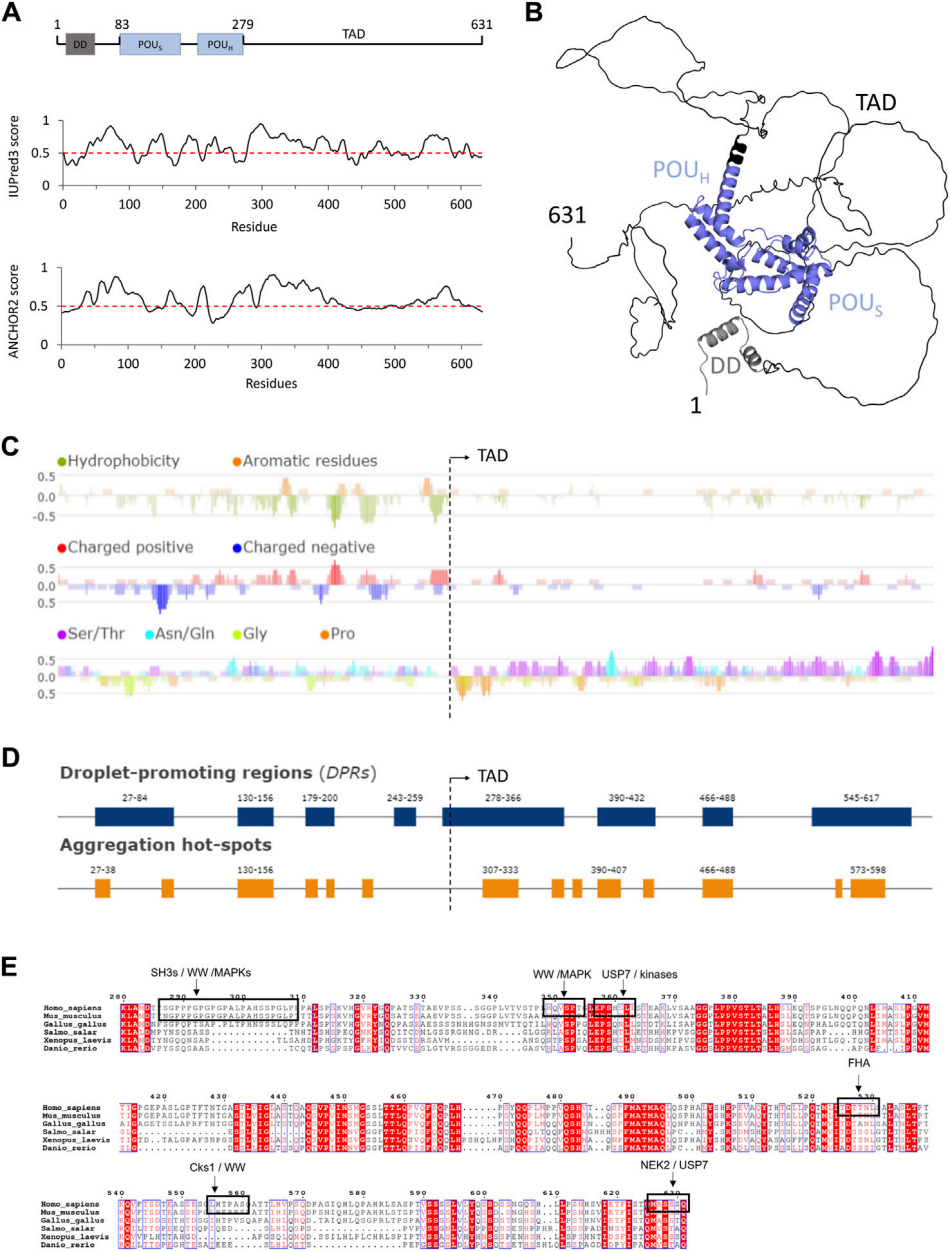
Structure prediction and sequence analysis of HNF-1A **(A)**. Top: Domain overview of full-length HNF-1A with residue numbers indicated above. DD—Dimerization domain. POU_S_—POU-specific domain. POU_H_—POU homeodomain. TAD—Transactivation domain. Middle: IUPred3 (Erdős et al., 2021) disorder prediction for full-length HNF-1A. Values above the threshold (red dashed line) indicate tendency for disorder, while values below the threshold indicate tendency for order. Bottom: ANCHOR2 (Mészáros et al., 2009) prediction of protein regions that undergo disorder-to-order transitions upon binding. High values represent highly disordered binding regions **(B)**. AlphaFold model for full-length HNF-1A (Jumper et al., 2021; Varadi et al., 2022). Coloring based on the domain presentation in **(A)**. **(C)** Fast Estimator of Latent Local Structure **(**FELLS) sequence analysis for full-length HNF-1A regarding residue hydrophobicity (top), amino acid charge (middle), and compositional bias towards Ser/Thr, Asn/Gln, Gly, and Pro residues (bottom). **(D)** FuzDrop predictions of droplet-promoting regions and aggregation hotspots in full-length HNF-1A. **(E)** Multiple sequence alignment of HNF-1A TAD (residues 280-631) for the model organisms *Homo sapiens*, *Mus musculus*, *Gallus gallus*, *Salmo salar*, *Xenopus laevis*, *Danio rerio*. Conserved residues are marked with red background, and similar residues are denoted in red notation. SLiMs retrieved from the ELM database are depicted by a black box and specified by the description above.

HNF-1A exhibits a multi-domain architecture typical for transcription factors (Figure 1A). The N-terminal dimerization domain (DD, residues 1–33) contains a helix-turn-helix motif and mediates homodimerization of HNF-1A or heterodimerization with the homologous protein HNF-1B (De Francesco et al., 1991; Rey-Campos et al., 1991; Narayana et al., 2006; Kind et al., 2022). The central α-helical DNA-binding domain (DBD, residues 83–279) is composed of a POU-specific domain (POU_S_) and a POU homeodomain (POU_H_), which together bind to the promoters of HNF-1A target genes (Chi et al., 2002). The C-terminal region of HNF-1A constitutes the transactivation domain (TAD, residues 280–631), which has remained undercharacterized. Three HNF-1A isoforms have been identified, which differ in the length of the TAD and exhibit varying levels of gene transactivation potentials (Bach and Yaniv, 1993).

Eukaryotic transcription factors commonly contain regulatory domains (Mitchell and Tjian, 1989; Staby et al., 2017), which can alter gene transcription in an activating or inhibiting manner, e.g., by binding to components of the transcriptional machinery or by mediating chromatin remodeling (Naar et al., 2001). These regulatory domains frequently contain intrinsically disordered regions (IDRs), which often exhibit a compositional sequence bias (Dyson and Wright, 2005; Staby et al., 2017). HNF-1A likely harbors such IDRs and several sites for protein-protein interactions in the C-terminal TAD. Specific regions within the TAD have been shown to promote transcriptional activity of HNF-1A (residue ranges 398–470, 544–631, and 440–506) and are thus proposed to function as activation domains (Toniatti et al., 1993; Bjørkhaug et al., 2005). However, detailed knowledge on the structural and functional features of the HNF-1A TAD is currently lacking, preventing a mechanistic understanding of the gene transcriptional functions of HNF-1A and the impact of *HNF1A* variants within this region.

Here, we present a bioinformatic approach to identify features in the TAD of HNF-1A that may be of functional importance in transcriptional regulation by HNF-1A. We further developed a protocol to recombinantly express and purify a TAD-containing HNF-1A protein and assessed the behavior of the TAD in solution. Finally, we present functional data supporting a liquid-liquid phase separation (LLPS) behavior of HNF-1A, which may play a role in gene transcriptional processes of pancreatic β-cells. This knowledge is important for the development of novel approaches in precision medicine and can lead to improved care for patients with HNF1A-MODY.

## 2 Materials and methods

### 2.1 Bioinformatic analyses

HNF-1A protein sequences from various model organisms were extracted from the UniProt (UniProt, 2021) database under the following accession codes: *Homo sapiens* (human): P20823, *Mus musculus* (mouse): P22361, *Gallus gallus* (chicken): Q90867, *Salmo salar* (Atlantic salmon): Q91474, *Xenopus laevis* (African clawed frog): Q05041, *Danio rerio* (zebrafish): Q8UVH4.

Disorder predictions of full-length human HNF-1A (*Hs*HNF-1A) were performed by using the IUPred3 (Erdős et al., 2021), ANCHOR2 (Mészáros et al., 2009) and the Fast Estimator of Latent Local Structure (FELLS) (Piovesan et al., 2017) webservers. The FELLS output was further utilized to investigate sequence composition, such as residue hydrophobicity, charge, and sequence compositional bias. AlphaFold (Jumper et al., 2021; Varadi et al., 2022) was employed for a tertiary structure prediction of full-length *Hs*HNF-1A. The predicted structure (AF-P20823-F1-model_v4.pdb) with residue specific predicted local distance difference test (pLDDT) scores and the corresponding predicted aligned error plot were retrieved from the AlphaFold Protein Structure Database (https://alphafold.ebi.ac.uk/entry/P20823). The FuzDrop algorithm (Hatos et al., 2022) was used to predict the tendency of *Hs*HNF-1A to undergo LLPS. A multiple sequence alignment of the HNF-1A TAD (*Hs*HNF-1A, residues 280–631) was generated using the Clustal Omega alignment tool (Sievers and Higgins, 2021; Madeira et al., 2022) and visualized using ESPript3.0 (Robert and Gouet, 2014). Potential short linear motifs (SLiMs) within the TAD were retrieved from the Eukaryotic Linear Motif (ELM) resource database (Kumar et al., 2022). The probability of HNF-1A to contain acidic activation domains was predicted by the “Predictor of Activation Domains using Deep Learning in Eukaryotes” (PADDLE) algorithm (https://paddle.stanford.edu).

### 2.2 Plasmid generation, recombinant protein expression, and purification

A mammalian pcDNA3.1/HisC expression vector harboring the cDNA sequence of full-length *Hs*HNF-1A (residues 1–631, UniProt ID P20823, isoform A, Ensembl transcript ID: ENST00000257555.11) (Bjørkhaug et al., 2003) was used to generate a bacterial expression construct for DBD-TAD (residues 83–631) by Gateway^®^ cloning technology (Katzen, 2007). Two PCR reactions were conducted in order to introduce an N-terminal Tobacco-Etch Virus protease site (ENLYFQG) and attB1/2 sites required for recombination. A BP reaction was performed to transfer the gene sequence into the pDONR221 entry vector (Invitrogen). A subsequent LR reaction was conducted for DNA insert transfer into the pTH27 (Hammarström et al., 2006) destination vector, generating a bacterial expression plasmid encoding for a His_6_-TEV-DBD-TAD construct (hereafter DBD-TAD). Sequence identity and integrity were verified by plasmid DNA sequencing. Primers used in cloning and sequencing are listed in Table 1.

**TABLE 1:** Primers used for the generation and sequencing of the His_6_-TEV-DBD-TAD expression plasmid.

Primer	Name/Target	Purpose
TCT​GAG​AAT​CTT​TAT​TTT​CAG​GGC​CCA​CCC​ATC​CTC​AAA​GAG​CTG​G	HNF-1A_83_FWD_TEV_part_attB1	Gateway cloning, PCR reaction 1
AGA​AAG​CTG​GGT​CTT​ACT​GGG​AGG​AAG​AGG​CCA​TCT​GG	HNF-1A_631_RVS_part_attB2	Gateway cloning, PCR reaction 1
GGG​GAC​AAG​TTT​GTA​CAA​AAA​AGC​AGG​CTC​TGA​GAA​TC	Complete_attB1_FWD	Gateway cloning, PCR reaction 2
GGG​GAC​CAC​TTT​GTA​CAA​GAA​AGC​TGG​GT	Complete_attB2_RVS	Gateway cloning, PCR reaction 2
TAA​TAC​GAC​TCA​CTA​TAG​GG	T7_init_FWD	Sequencing of pTH27 expression plasmid
GCT​AGT​TAT​TGC​TCA​GCG​G	T7_term_RVS	Sequencing of pTH27 expression plasmid
GCCCGATGGTCATGAC	HNF1A_n1252_RVS	Sequencing of pTH27 expression plasmid

DBD-TAD was recombinantly expressed in *Escherichia coli* Rosetta (DE3). Bacteria were cultured in LB medium at 37°C until an OD_600_ value of 0.6–0.8 was reached. Protein expression was induced by the addition of 1 mM IPTG and conducted at 18°C for 20 h. Bacteria were harvested by centrifugation (6,000 × *g*, 10 min, 4°C) and resuspended in lysis buffer containing urea as denaturation reagent (50 mM Tris pH 8.5, 500 mM NaCl, 6 M urea, 10 mM imidazole, 1 mM DTT, 1 mM PMSF, 1× cOmplete EDTA-free protease inhibitor cocktail). The sample was ultrasonicated (7 min, 1 s on/off cycles, 25 W), and the lysate was clarified by centrifugation (16,000 × *g*, 30 min, 4°C).

The soluble fraction was loaded onto a pre-equilibrated Ni-NTA column with a 2-mL bed volume. The column was washed with 15 column volumes (CVs) of wash buffer (50 mM Tris pH 8.5, 500 mM NaCl, 6 M urea, 10 mM imidazole, 1 mM DTT, 1 mM PMSF), before elution was performed using a stepwise imidazole gradient (40 mM, 60 mM, 80 mM, 300 mM imidazole) and a fraction volume of 3 CVs. Fractions containing the protein of interest were pooled and dialyzed into a buffer lacking urea (20 mM Tris pH 8.5, 500 mM NaCl, 1 mM DTT), using dialysis tubing with a 12–14 kDa molecular weight cut-off (Spectra/Por). The dialyzed sample, containing the refolded His-tagged DBD-TAD protein, was concentrated and subjected to size exclusion chromatography (SEC). A Superdex 200 10/300 GL Increase column (GE Healthcare) was used at a flow rate of 0.5 mL/min with a running buffer containing 20 mM Tris pH 8.5, 500 mM NaCl, 1 mM TCEP. Pooled SEC fractions containing His-tagged DBD-TAD were concentrated, snap-frozen in liquid N_2_, and stored at −80°C.

The DBD of HNF-1A (residues 83–279) was recombinantly expressed and purified as previously described (Kind et al., 2022) and used for control purposes.

### 2.3 Synchrotron radiation circular dichroism

SRCD experiments were performed on the AU-CD beamline, ASTRID2 synchrotron (Aarhus, Denmark). DBD and DBD-TAD were dialyzed into SRCD buffer (20 mM Na phosphate pH 7.8, 150 mM NaF, 1 mM TCEP) prior to measurements. Samples with various 2,2,2-trifluoroethanol (TFE) concentrations were prepared by mixing with 100% TFE (Sigma-Aldrich T63002). All samples were measured at an HNF-1A concentration of 0.5 mg/mL, except for DBD-TAD in the presence of 70% TFE, where the HNF-1A concentration was 0.45 mg/mL. SRCD measurements were performed at 10°C, using 0.1 mm quartz cuvettes (Hellma Analytics). Three wavelength scans per sample were recorded, applying a scan range of 170–280 nm with a 1-nm step size. Data processing was done using Microsoft Excel and CDtoolX (Miles and Wallace, 2018). Spectrum deconvolution was performed by using the BeStSel algorithm (Micsonai et al., 2015; Micsonai et al., 2018) with a spectral range of 180–250 nm (https://bestsel.elte.hu/index.php) and by using the DichroIDP program with the IDP175 reference dataset (Miles et al., 2023). SRCD datasets can be accessed via zenodo.org (DOI: 10.5281/zenodo.8328701) and in the PCDDB (Ramalli et al., 2022) under the series codes CD0006461 (HNF-1A DBD-TAD) and CD0006462 (HNF-1A DBD).

### 2.4 Small-angle X-ray scattering

Synchrotron SAXS experiments for DBD-TAD were performed on the CoSAXS beamline (Kahnt et al., 2021), MAX IV Laboratory (Lund, Sweden). DBD-TAD was measured at ∼2.5 mg/mL in batch mode (4 mM Tris pH 8.5, 100 mM NaCl, 1 mM TCEP). Measurements were performed at 10°C at a wavelength of 1 Å (12.4 keV). Data were collected over an angular range of 3.8 × 10^−4^–6.4 × 10^−1^ Å^−1^ with 300 frames at an exposure time of 20 ms per frame. Final sample and buffer scattering curves were obtained by averaging the frames that matched each other to avoid incorporation of radiation damage.

Data reduction and analysis were done using the ATSAS 3.2.1 package (Manalastas-Cantos et al., 2021). Analyses of the Guinier region, the particle distribution function p(r), and molecular weight estimates were calculated using PRIMUSqt. Additional molecular weight estimation was performed using the SAXS MoW 2.0 method (http://saxs.ifsc.usp.br, integration upper limit q_max_ = I(0)/I(q_max_) = 10^2.25^) as well as by using the Debye formalism, essentially as described in (Raasakka et al., 2019) and according to original methodology (Calmettes et al., 1994; Fitzkee and Rose, 2004; Bernadó and Blackledge, 2009).

The ensemble optimization method (EOM) algorithm was used to generate an ensemble of models satisfying the SAXS data (Bernadó et al., 2007; Tria et al., 2015). The crystal structure of the DBD [PDB: 1IC8, (Chi et al., 2002)] was used to provide rigid bodies for POU_S_ (residues 87–180) and POU_H_ (residues 209–276). The EOM algorithm was run for native-like behavior of disordered regions, with the POU_S_ domain being fixed in position. The EOM conformers were visualized using PyMOL (DeLano, 2002).

Parameters used in SAXS data collection, analysis, and modelling for both the DBD-TAD and the previously published DBD dataset (Kind et al., 2022) are listed in Supplementary Table S1. SAXS datasets were submitted to the SASBDB (Valentini et al., 2015) and are available under the accession codes SASDS29 (HNF-1A DBD-TAD) and SASDSZ8 (HNF-1A DBD).

### 2.5 Differential interference contrast microscopy

Purified DBD-TAD and DBD were diluted in storage buffer (20 mM Tris pH 8.5, 500 mM NaCl, 1 mM TCEP), yielding a final concentration of 10–25 μM. PEG8000 was added to a final concentration of 10% w/v. For each protein concentration, a 10-µL sample was prepared on a microscopy slide and differential interference contrast (DIC) images were acquired using a Zeiss Axiovert 200M wide-field fluorescence microscope equipped with a LD Plan-NEOFLUAR 40×/0.6 Ph2 objective and an AxioCam HR camera. Image acquisition was performed using the AxioVision software (Carl Zeiss, version 4.5).

### 2.6 Immunofluorescence microscopy

MIN6 cells (Miyazaki et al., 1990; Poitout et al., 1996), kindly provided by Professor Claes Wollheim (Lund University, Sweden), were cultured in DMEM growth medium (Gibco), supplemented with 15% fetal bovine serum and 1% v/v penicillin-streptomycin (Sigma-Aldrich P4333). Cells were incubated at 37°C and 5% atmospheric CO_2_ in a humidity incubator. For immunofluorescence (IF), MIN6 cells were seeded on coverslips in 24-well plates. IF staining was performed at room temperature (RT). 24 h post-seeding, cells were washed twice with phosphate-buffered saline (PBS) and fixed by incubation in a phosphate buffer containing 3% paraformaldehyde (Sigma-Aldrich) for 25 min. Samples were washed three times with PBS, permeabilized in 0.1% Triton X-100 (Sigma-Aldrich) in PBS for 10 min, and washed three times with PBS. Binding epitopes were blocked by incubation with 2% normal goat serum (GS, Invitrogen)/8% bovine serum albumin (BSA, Sigma-Aldrich) in PBS for 1 h, followed by three PBS wash steps. Primary anti-HNF-1A antibody (Invitrogen, PA5-83263) was applied for 1 h, using a 1:100 dilution in 2% GS/8% BSA-PBS. Samples were washed three times and stored in PBS overnight at 4°C. Secondary antibody [Alexa Fluor^®^ 488 AffiniPure Goat Anti-Rabbit IgG (H + L), Jackson ImmunoResearch, 111-545-003] was applied for 45 min, using a 1:100 dilution in 2% GS/8% BSA-PBS. Samples were washed three times with PBS and mounted on glass slides using ProLong™ Diamond Antifade DAPI mountant (Invitrogen, P36962). Mounting solution was allowed to solidify overnight, and the slides were inspected on the following day. Samples were imaged using a Zeiss Axiovert 200M wide-field fluorescence microscope equipped with an AxioCam HR camera, a Plan-NEOFLUAR 100×/1.30 Ph3 oil immersion objective and filters appropriate for the detection of DAPI and GFP/Alexa-488 signals. Images were acquired by using the AxioVision software (Carl Zeiss, version 4.5) and superposed using the Fiji ImageJ software (Wayne Rasband, National Institutes of Health, United States, version 1.52a) (Schindelin et al., 2012).

### 2.7 Droplet simulation protocol

Molecular dynamics simulations of the LLPS behavior of HNF-1A TAD (residues 280–631) were performed using the 1 bead per amino acid (1BPA) molecular dynamics model (Ghavami et al., 2013; Ghavami et al., 2014), with updated parameters described in (Driver et al., 2022). The initial cubic simulation box is populated with molecules (using a random initial conformation) with their center of mass placed upon a regular grid, with a small buffer region to avoid overlap between molecules. All simulations are carried out at a temperature of 300 K, 150 mM ion concentration (*κ* = 1.27 nm^−1^), and use a timestep of 20 fs. For equilibration of the droplet, energy minimization on the initial configuration is used (energy tolerance of 1 kJ mol^−1^ nm^−1^), before 50 ns NVT Langevin dynamics simulations (Nosé-Hoover thermostat with τ_t_ = 100 ps), followed by 500 ns NPT Langevin dynamics (Nosé-Hoover thermostat with τ_t_ = 100 ps and a Berendsen barostat with τ_p_ = 10 ps, 1 bar reference pressure and a compressibility of 4.5 10^−5^ bar^−1^). The end state of the NPT equilibration step is inserted into a new periodic box with a volume chosen to give a total residue density of 80 mM, after recentering on the center of mass and after the molecules have been unwrapped across the previous periodic boundary conditions. A second energy minimisation step is applied in the new simulation box to relax the molecules after the box expansion (energy tolerance of 1 kJ mol^−1^ nm^−1^). A final 3 μs NVT equilibration/production run (Nosé-Hoover thermostat with τ_t_ = 100 ps) is used for data collection. The trajectory is sampled every 5 ns to determine whether convergence was reached.

## 3 Results and discussion

### 3.1 Structure prediction of HNF-1A

Bioinformatic sequence analyses provided insights into structural features and sequence characteristics of human HNF-1A (Figure 1). Disorder prediction using IUPred3 was performed to analyze the residue-dependent propensity of HNF-1A to fold into globular domains or remain disordered (Figure 1A) (Erdős et al., 2021). While the α-helical DD and DBD of HNF-1A were predicted to fold, the linker between DD and DBD showed a high tendency to remain disordered (Figure 1A). The flexibility of this linker region has previously been demonstrated by SAXS (Kind et al., 2022), illustrating the agreement between *in vitro* experiments and *in silico* prediction. The IUPred3 prediction indicated that the TAD likely contains long IDRs. Disorder was predicted for the residue ranges 280–420, 460–500, and 540–590, while the regions harboring residues 420–460, 500–540, and 590–631 may fold in a context-specific manner (Figure 1A). A complementary analysis using the ANCHOR2 algorithm was in agreement with the IUPred3 prediction (Figure 1A). The residue ranges 300–400 and 550–600 were predicted to contain disordered binding sites that can undergo disorder-to-order transitions upon binding to a partner protein (Figure 1A).

Disordered protein regions harbor intrinsic flexibility, which leads to a dynamic and non-globular protein conformation. We found that tertiary structure predictions of HNF-1A using AlphaFold (Jumper et al., 2021; Varadi et al., 2022) were in agreement with disorder predictions by IUPred3 and ANCHOR2 (Figure 1B). The per-residue estimate of prediction confidence (pLDDT score) was <50 for residues 300–631, indicating a disordered conformation for this protein region (Supplementary Figures S1A, B). Apart from a C-terminal extension of helix α8 in the DBD, the AlphaFold prediction suggested an entirely disordered TAD (Figure 1B; Supplementary Figure S1B). The model presented in Figure 1B depicts the TAD in one possible conformation which co-exists with numerous other conformations that are sampled by the TAD. This conformational flexibility is reflected in the predicted aligned error analysis (Supplementary Figure S1C), which indicated that the prediction confidence for the relative position of two given HNF-1A residues was high within the folded domains (DD, POU_S_, POU_H_), but low for residues within the TAD.

It should be noted that the AlphaFold model may not represent context-dependent structural changes of the TAD. According to the ANCHOR2 prediction (Figure 1A), specific segments of the TAD are likely to undergo disorder-to-order transitions when binding to other molecules. Depending on the interaction interface, these specific segments may undergo a transition from a coil-like secondary structure to an α-helical structure or a β-strand conformation to maximize intermolecular hydrophobic interactions when binding to the interaction partner (Dyson, 2013). Folding events, which follow such a coupled folding and binding mechanism, have been demonstrated for IDRs in other transcription factors (e.g., DREB2A, p53, HIF1α) when binding to specific protein partners (Dyson and Wright, 2005; Oldfield et al., 2008; O’Shea et al., 2017).

The structure predictions by IUPred3, ANCHOR2, and AlphaFold indicated that the HNF-1A TAD is predominantly disordered. As intrinsic protein disorder is critical for eukaryotic gene transcriptional regulation (Liu et al., 2006; Mar et al., 2023), the predictions of TAD disorder underline its potential role in the gene transcriptional activity of HNF-1A. A disordered conformation of the TAD may be important for HNF-1A to engage in the formation of macromolecular complexes, as it may allow HNF-1A to sample a large physical space and to simultaneously bind several other transcription factors and co-activators. The ability of the TAD to fold in a context-specific manner could provide versatility in protein-protein interactions of HNF-1A, which may be important for the response to different signals in different cellular states. Besides mediating the binding to other proteins, the intrinsically disordered TAD may contribute to DNA recognition and binding, thereby modulating the gene transcriptional activity of HNF-1A (Mar et al., 2023).

### 3.2 Sequence composition of the TAD

A general feature of intrinsically disordered proteins (IDPs) is the absence of hydrophobic clusters and an enrichment of charged and polar residues (Uversky, 2002). Indeed, a hydrophobicity analysis using FELLS illustrated the lack of hydrophobic residues in the TAD compared to the globular DD and DBD in the N-terminal half of HNF-1A (Figure 1C). Interestingly, charge distribution analysis of the HNF-1A sequence revealed that the TAD contains very few charged residues (Figure 1C). This observation was surprising, as charged amino acids generally promote solubility and are frequently found in IDPs (Uversky, 2002). However, the sequence analysis also revealed that the TAD is highly enriched in Ser and Thr residues. Such a polar tract may facilitate the interaction with the polar environment and promote a disordered state of the TAD (Figure 1C).

In addition to modulating protein solubility, the side chains of Ser and Thr residues can be phosphorylated, leading to changes in protein function (Day et al., 2016). The high prevalence of Ser and Thr residues in the TAD of HNF-1A may indicate that it is a target of post-translational modification by kinases and phosphatases. As a transcription factor, HNF-1A may be phosphorylated by kinases within the basal transcriptional machinery, e.g., cdk7-9, TAFII250, or TFIIF (Riedl and Egly, 2000). Cdk7 phosphorylates the activation domains of the transcription factors ERα, E2F-1, and p53, which results in changes in their transactivating properties (Riedl and Egly, 2000). In line with the above, phosphorylation of TAD residues may dynamically affect the ability of HNF-1A to interact with transcriptional co-activators/repressors and chromatin remodeling proteins by altering the protein interaction interface, ultimately leading to changes in the transactivation potential of HNF-1A. Alternatively, side chain phosphorylation of the TAD may modulate the recognition sites for specific adapter proteins, which could lead to changes in HNF-1A localization (Day et al., 2016). Finally, TAD phosphorylation may create or disturb binding sites for the components of the ubiquitination machinery, which could lead to changes in HNF-1A protein turnover due to altered proteasomal degradation (Hunter, 2007).

Transcription factors contain specific activation domains, which are crucial for the recruitment of co-activators and the formation of the pre-initiation complex at specific promoter target sites (Frietze and Farnham, 2011). The most reported activation domains in transcription factors are acidic activation domains (AADs), which require a balance between acidic and hydrophobic residues (Sanborn et al., 2021; Staller et al., 2022). The suggested “acidic exposure model” of AAD function describes that hydrophobic residues are interspersed with acidic residues, thereby preventing a hydrophobic collapse of the protein region and promoting exposure of the hydrophobic residues for interaction with co-activator protein surfaces. Based on these observations, the “Predictor of Activation Domains using Deep Learning in Eukaryotes” (PADDLE) algorithm was developed to identify such segments in protein sequences (Sanborn et al., 2021). When applying PADDLE to the HNF-1A sequence, we found that the protein does not contain any regions with an amino acid composition indicative for an AAD (Supplementary Figure S2). The transactivation potential of the TAD may thus unfold via a different mechanism. Considering the sequence composition of the TAD (Figure 1C), we speculate that activation domains rich in Gln, Ser, and Pro may play a role in the function of the HNF-1A TAD. Only a limited number of such alternative activation domains have been annotated thus far, such as the Glu and Pro rich activation domains in the POU homeodomain protein Oct-2 (Tanaka et al., 1994) and a Ser rich activation domain in the v-Rel protein of the Rel/NF-kappaB transcription factor family (Chen et al., 1999). Expanding on the current knowledge of activation domain characteristics, a recently reported large-scale mapping approach identified novel activation domains in human transcriptional effector domains by testing transcription factor fragments (tiles) in a cell-based reporter assay (DelRosso et al., 2023). Several identified activation domains were composed of hydrophobic residues interspersed with acidic, Glu, Pro, and/or Ser residues (DelRosso et al., 2023). Employing a quantitative cell-based reporter screen with defined HNF-1A TAD fragments, as demonstrated by Sanborn et. al (2021), will aid in the identification of HNF-1A activation domains and contribute to the understanding of alternative activation mechanisms besides the AAD model.

In recent years, researchers have uncovered the ability of transcription factors to activate gene transcription by undergoing LLPS. The formation of transcriptional condensates is often mediated via activation domains and leads to the dynamic compartmentalization of the gene transcriptional machinery, including RNA polymerase II and its co-factors (Boija et al., 2018; Sabari, 2020). We hypothesized that the HNF-1A TAD may have the potential to activate gene transcription in the same way. We utilized the FuzDrop algorithm to compute the probability of HNF-1A to undergo spontaneous LLPS (Hatos et al., 2022), and the obtained pLLPS score of 0.9932 suggested a strong LLPS tendency. The computed droplet-promoting regions (DPRs) were found across the entire HNF-1A sequence (Figure 1D), whereby long DPRs were localized to the intrinsically disordered DD-DBD linker (residues 27–84) and the HNF-1A TAD (residue ranges 278–366, 390–432, and 545–617). The prediction is in line with the observation that LLPS often involves weak, multivalent interactions between IDRs (Dignon et al., 2020). Interestingly, some of the predicted DPRs overlapped with two annotated activation domains in the TAD (residue ranges 398–470 and 544–631), which may hint towards a transactivation mechanism involving LLPS (Toniatti et al., 1993; Bjørkhaug et al., 2005). In cases of LLPS dysregulation, protein condensates can develop into a transient gel and ultimately evolve into an amyloid state of protein aggregates, which is usually driven by characteristic cross-β-structures (Vendruscolo and Fuxreiter, 2022). Such irreversible amyloid states can lead to cytotoxicity and consequently cause various diseases, such as cancer, neurodegeneration, and infectious diseases (Alberti and Dormann, 2019; Mathieu et al., 2020). The switch between a droplet and amyloid state involves protein regions which are able to adopt both a disordered droplet-promoting and an ordered aggregate-promoting binding mode. Computational predictions indicated that in 87% of the human proteome, DPRs and amyloid-promoting regions co-occur in the same sequence region (Vendruscolo and Fuxreiter, 2022). In accordance with this observation, the FuzDrop algorithm predicted that some of the DPRs in HNF-1A contain aggregation hotspots, which can initiate the irreversible maturation of droplets and thus lead to protein aggregation (Figure 1D). Future studies may reveal the relevance of the predicted DPRs in HNF-1A and the potential impact of droplet aggregation upon dysregulation of β-cell function, e.g., in type 2 diabetes, or alteration of the HNF-1A protein sequence, e.g., in HNF1A-MODY.

### 3.3 Sequence conservation and short linear motifs in the TAD

We generated a multiple sequence alignment to investigate the sequence conservation of the TAD (Figure 1E). Some TAD regions are highly conserved across species, such as residue ranges 374–387, 457–469, and 487–496, while conservation in other regions is mainly restricted to mammals, e.g., in residue ranges 285–311, 330–350, and 571–589.

IDRs frequently harbor SLiMs, which are sequence elements of 6–12 residues that mediate the binding to folded interaction partners or promote LLPS by partaking in multivalent interactions (Bugge et al., 2020). In order to identify potential SLiMs in the TAD of HNF-1A, we performed a search in the ELM resource (Kumar et al., 2022). High-confidence SLiMs are marked in the multiple sequence alignment (Figure 1E) and include interaction sites for WW domains (residues 287–308, residues 555–560), SH3 domains (residues 287–308), FHA domains (residues 525–530), as well as a recognition site for USP7 ubiquitination enzymes (residues 626–631). Interestingly, many of these SLiMs overlapped with recognition sites for different kinases, such as MAPK, Cks1, and NEK2, which may promote Ser or Thr phosphorylation within the motifs and thereby alter their ability to interact with the respective protein domains. The predicted SLiMs were localized in both highly conserved and less conserved regions (Figure 1E).

In conclusion, our sequence analyses provided a useful starting point for the functional dissection of the HNF-1A TAD. Experimental studies will be required in the future to investigate the biological roles of specific TAD regions and their dynamic regulation. Biochemical and structural approaches will shed light on the structure-function relationships of HNF-1A in solution.

### 3.4 Recombinant expression and purification of a TAD-containing HNF-1A protein

In order to provide a basis for functional studies, we set out to biophysically and structurally characterize the TAD of HNF-1A. As full-length HNF-1A had not been characterized *in vitro* prior to this study, we designed several TAD-containing expression constructs in order to cover all defined HNF-1A domains, and, at the same time, provide flexibility in case of expression/solubility problems caused by certain domains. We thus generated three N-terminally His_6_-tagged HNF-1A expression constructs harboring the TAD (DD-DBD-TAD, residues 1–631; DBD-TAD, residues 83–631; TAD, residues 280–631), whereby the information on the DBD and TAD boundaries was acquired from a published X-ray crystal structure of DNA-bound DBD [PDB ID: 1IC8, (Chi et al., 2002)]. The N-terminal His_6_-tag was chosen as a simple strategy to allow for Ni-NTA affinity chromatography-based purification of recombinant HNF-1A.

Initial overexpression in Rosetta (DE3) resulted in insoluble protein, which may be functionally connected to the predicted disordered nature of the TAD (Figures 1A, B) or the tendency to form condensates or amyloid-like protein aggregates (Figure 1D). In order to enhance the solubility of the proteins, we screened for optimal expression and lysis conditions. Following established strategies to improve protein folding and to reduce protein aggregation (Sørensen and Mortensen, 2005; Leibly et al., 2012), we varied expression systems [Lemo21 (DE3) *E. coli*, Sf9 *Spodoptera frugiperda*], expression conditions (1–72 h, 20/37°C), and lysis buffer compositions (screening for NaCl concentrations, pH value, and stabilizing additives). However, none of these adjustments yielded soluble protein (data not shown). Future attempts to improve the soluble expression of HNF-1A may involve a fusion to the *Bombyx mori* 30Kc19 protein, which has been proven useful for the expression of otherwise unstable transcription factors, e.g., Oct-4, Sox2, and Klf4 (Ryu et al., 2016).

**FIGURE 2:**
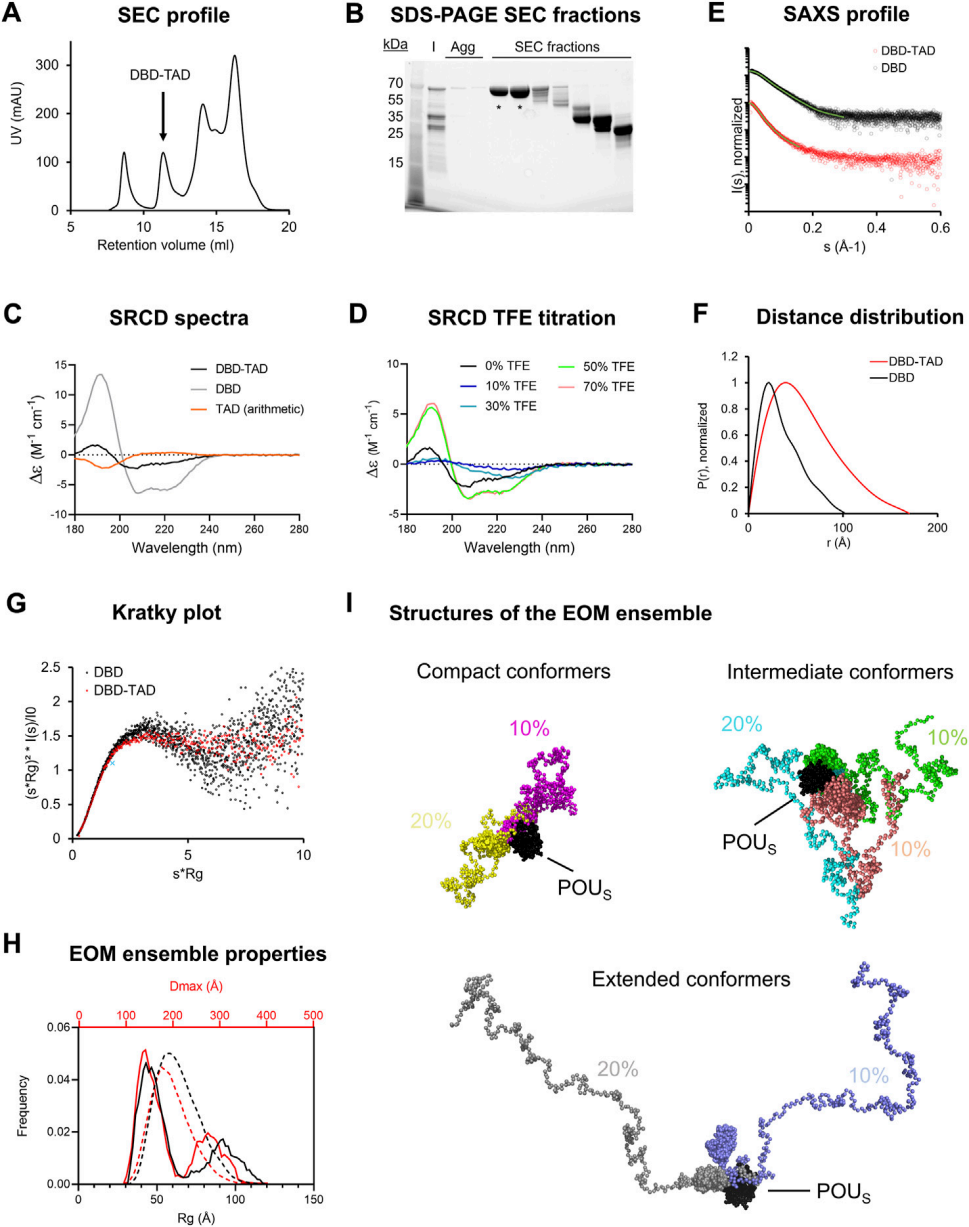
Purification and biophysical characterization of the DBD-TAD construct **(A)**. SEC profile with the DBD-TAD containing peak indicated **(B)**. SDS-PAGE analysis of respective SEC fractions from various peaks of the chromatogram shown in **(A)**. Agg—SEC fractions containing aggregated proteins. The bands marked with “*” were pooled and used for experiments. Lower molecular weight species in the residual SEC fractions corresponded to degradation products of the recombinant protein **(C)**. SRCD spectra for DBD (grey) and DBD-TAD (black). A theoretical spectrum for the isolated TAD (orange) was calculated by subtraction of the two obtained spectra **(D)**. TFE titration experiment for DBD-TAD **(E–G)**. SAXS data analyses for DBD-TAD. DBD SAXS data, previously published by us (Kind et al., 2022), are presented for direct comparison **(E)**. Scattering curve **(F)**. Distance distribution function **(G)**. Normalized Kratky plot, with the cross indicating the expected maximum for a globular particle (√3, 1.104) (Durand et al., 2010) **(H)**. *D*_max_ (red) and *R*_*g*_ (black) distributions from EOM analysis for DBD-TAD, illustrating ensemble frequencies as solid lines and pool frequencies as dashed lines **(I)**. Seven conformer structures in the generated EOM ensemble with assigned abundance values, each represented in a different color. The models were superposed based on the POU_S_ domain (black). Conformers are grouped by the degree of compaction.

We proceeded with protein purification from the insoluble lysate fraction, whereby we focused on the DBD-TAD construct due to relatively high expression levels compared to the other two tested constructs (DD-DBD-TAD, TAD). We solubilized the protein under denaturing conditions during bacterial lysis and performed an initial purification step using Ni-NTA affinity chromatography. The protein did not have a strong affinity towards the Ni-NTA material, as relatively low imidazole concentrations (40–80 mM) were sufficient for elution. Moreover, the elution fraction contained three strong bands, indicating a C-terminal truncation of overexpressed DBD-TAD. In order to refold the denatured protein into its native form, we dialyzed the elution fractions into buffer lacking denaturant. We performed a final SEC step to separate monomeric DBD-TAD from aggregates and suspected degradation products (Figures 2A, B). Mass spectrometry confirmed that both the purified protein and the suspected degradation products were derived from human HNF-1A. The degradation from the C-terminus may be explained by the expected disordered nature of the TAD (Figures 1A, B), as degradation motifs might be exposed and can be recognized by bacterial proteases. The degradation of DBD-TAD may already take place during the protein expression step; however, additional degradation might occur during protein purification. In the future, this problem could potentially be solved by the introduction of a C-terminal protein tag acting as protective chaperone, e.g., maltose-binding protein (MBP), N-utilizing substance A (NusA), small ubiquitin related modifier (SUMO), or glutathione S-transferase (GST). In particular, MBP and SUMO exhibit the useful property of promoting the translocation of the recombinantly expressed fusion protein from the cytosol to cellular locations with lower protease contents, such as the cell membrane and the nucleus, respectively (Butt et al., 2005; Costa et al., 2014).

Despite the observed protein degradation, we were able to isolate a pure protein that exhibited a migratory behavior expected for the theoretical molecular weight of DBD-TAD (62 kDa, Figure 2B). As our goal was to biophysically characterize a recombinant protein that contained the entire TAD, it was important to retain a homogenous sample without degradation products. Hence, to avoid the gradual formation of DBD-TAD degradation products over time, we aimed to conduct all characterization experiments immediately after purification.

### 3.5 The HNF-1A TAD is intrinsically disordered as revealed by SRCD and SAXS

SRCD experiments were performed to assess the secondary structure content of the purified protein. The SRCD spectra indicated that DBD-TAD contained both α-helical structures and random-coil regions (Figure 2C). In order to quantify the secondary structure elements within the protein, we performed a deconvolution of the SRCD spectra (Table 2). Calculations with the BeStSel algorithm (Micsonai et al., 2015; Micsonai et al., 2018) revealed that DBD-TAD contained 12% α-helical structures, 26.9% β-sheet, 17.5% turns, and 43.6% other structures, e.g., bends, loops, and irregular or invisible structure elements (Table 2). As we expected DBD-TAD to contain a large fraction of disordered regions, we also employed the recently developed DichroIDP program, which uses a novel protein reference dataset (IDP175) that is suitable for analyses of proteins containing significant amounts of disordered structure (Miles et al., 2023). DichroIDP calculated a DBD-TAD composition of 4% α-helical structures, 39% β-sheet, 30% turns, and 27% disordered regions (Table 2). The deconvolution with both algorithms indicates that a major part of DBD-TAD is intrinsically disordered, which confirms the secondary structure predictions from IUPred3, ANCHOR2, and AlphaFold (Figures 1A, B). The α-helical CD signals likely arise from the folded POU_S_ and POU_H_ domains in the DBD (Chi et al., 2002; Kind et al., 2022). The considerable amount of β-sheet content could correspond to amyloid-promoting disordered regions which may adopt transient β-like formations, as predicted by the FuzDrop algorithm (Figure 1D). We compared DBD-TAD with a TAD-deficient DBD protein (Figure 2C). The SRCD spectrum of DBD indicated that the protein predominantly contained α-helices (Figure 2C), which was confirmed by the spectrum deconvolution (Table 2) and in agreement with previous studies (Narayana et al., 2001; Kind et al., 2022). Since DBD-TAD contained both α-helical and disordered regions, and the isolated DBD produced an SRCD spectrum indicative of a high α-helical structure content (Figure 2C), we concluded that the TAD of HNF-1A likely adopts a random-coil structure in solution. We performed a subtraction of the DBD-TAD and DBD spectra, yielding a theoretical TAD spectrum, which indicated a disordered protein conformation and confirmed our hypothesis (Figure 2C). A deconvolution of this theoretical TAD spectrum confirmed that the TAD predominantly consisted of turns, disordered regions, and β-sheet structures (Table 2). As noted above, the β-sheet content may correspond to transient β-sheet structures adopted by the predicted amyloid-promoting regions in the TAD (Figure 1D). To test the propensity of the TAD to fold into an α-helical structure, we performed a TFE titration experiment, in which α-helical structure of the DBD-TAD construct was induced by the addition of ≥50% TFE (Figure 2D; Table 2). Notably, intermediate TFE concentrations (10%–30%) decreased the SRCD signal across the entire wavelength range (Figure 2D), which may be due to secondary processes causing light scattering, such as LLPS or aggregation.

**TABLE 2:** SRCD spectra deconvolution by BeStSel (Micsonai et al., 2015; Micsonai et al., 2018) and DichroIDP (Miles et al., 2023) algorithms.

Sample	Helix (%)	β-sheet (%)	Turns (%)	Others/Disorder (%)
DBD-TAD				
BeStSel algorithm	12	26.9	17.5	43.6
DichroIDP IDP175	4	39	30	27
DBD				
BeStSel algorithm	54.8	3	8.3	33.8
DichroIDP IDP175	53	11	18	19
TAD (arithmetic)				
BeStSel algorithm	0	38.5	22.3	39.2
DichroIDP IDP175	5	37	27	30
DBD-TAD 10% TFE				
BeStSel algorithm	1.6	31.4	20.9	46.1
DichroIDP IDP175	15	36	19	30
DBD-TAD 30% TFE				
BeStSel algorithm	5.6	24.6	22.2	47.6
DichroIDP IDP175	15	36	19	30
DBD-TAD 50% TFE				
BeStSel algorithm	26	14.5	16.8	42.7
DichroIDP IDP175	34	13	29	22
DBD-TAD 70% TFE				
BeStSel algorithm	28.1	14.5	15.8	41.5
DichroIDP IDP175	46	4	29	20

We employed SAXS to study the molecular dimensions and shape of DBD-TAD (Figures 2E–G). We compared the scattering data to a published SAXS dataset for the isolated DBD (Kind et al., 2022), allowing us to study the contributions of the HNF-1A TAD. The DBD-TAD sample was free of aggregation (Figure 2E). Molecular weight analysis resulted in differing molecular weight estimates depending on the algorithm used (Supplementary Table S1). The molecular weight estimates based on the V_c_ and the SAXSMoW methods, which are suitable approaches for the analysis of intrinsically disordered proteins (Rambo and Tainer, 2013; Piiadov et al., 2019), indicated that DBD-TAD was present in monomeric state (Supplementary Table S1). The radius of gyration (*R*_*g*_) of DBD-TAD was determined using Guinier analysis (*R*_*g*_ = 4.9 nm) and the Debye formalism (*R*_*g*_ = 5.1 nm). The molecular dimensions of DBD-TAD were also analyzed using the paired distance distribution function (*R*_*g*_ = 4.8 nm, *D*_max_ = 17 nm). DBD-TAD presented larger *R*_*g*_ and *D*_max_ compared to the isolated DBD [*R*_*g*_ = 2.8 nm, *D*_max_ = 10 nm, Supplementary Table S1, (Kind et al., 2022)], which was expected due to the higher molecular weight of DBD-TAD. The dimensionless Kratky plot indicated that both DBD and DBD-TAD were highly flexible and had a non-globular shape (Figure 2G), which is likely due to the linker region between the POU_S_ and POU_H_ domain and the disordered TAD. Based on the scattering data, we generated a 3-dimensional model for DBD-TAD to visualize its molecular shape (Figure 2H). We utilized EOM to generate an ensemble of conformers that together satisfy the SAXS data (Supplementary Table S1; Figure 2H). A published crystal structure of a DBD:DNA complex [PDB: 1IC8 (Chi et al., 2002)] was used to model POU_S_ and POU_H_ as globular domains. Missing residues were modeled as flexible regions. The data were satisfied by an ensemble containing seven conformers with approximately equal abundance, exhibiting an average *R*_*g*_ of 64 Å and a *D*_max_ of 198 Å. An analysis of the ensemble properties revealed a bi modal distribution of the *R*_*g*_ and *D*_max_ parameters (Figure 2H), with one population exhibiting a peak *R*_*g*_ of ∼45 Å and a peak *D*_max_ of ∼140 Å, and another population having larger molecular dimensions with a peak *R*_*g*_ of ∼90 Å and a peak *D*_max_ of ∼280 Å. This is reflected in the generated conformer models, in which the TAD is present in compact, intermediate, and extended conformations (Figure 2I).

The SRCD and SAXS analyses confirmed the predicted intrinsic disorder of the HNF-1A TAD. The employed methods are, however, limited in their information content. In order to obtain data at atomic resolution, the biophysical analysis could be extended by the application of nuclear magnetic resonance (NMR) spectroscopy. This technique has been employed for numerous transcription factors, e.g., p53, human glucocorticoid receptor, and radical-induced cell death 1, and helped deconvolute the structure of TADs as well as their role in DNA recognition and IDR-mediated protein binding (Arai et al., 2012; Kim et al., 2017; Krois et al., 2018; Staby et al., 2021).

### 3.6 The TAD promotes LLPS of HNF-1A *in vitro*

**FIGURE 3:**
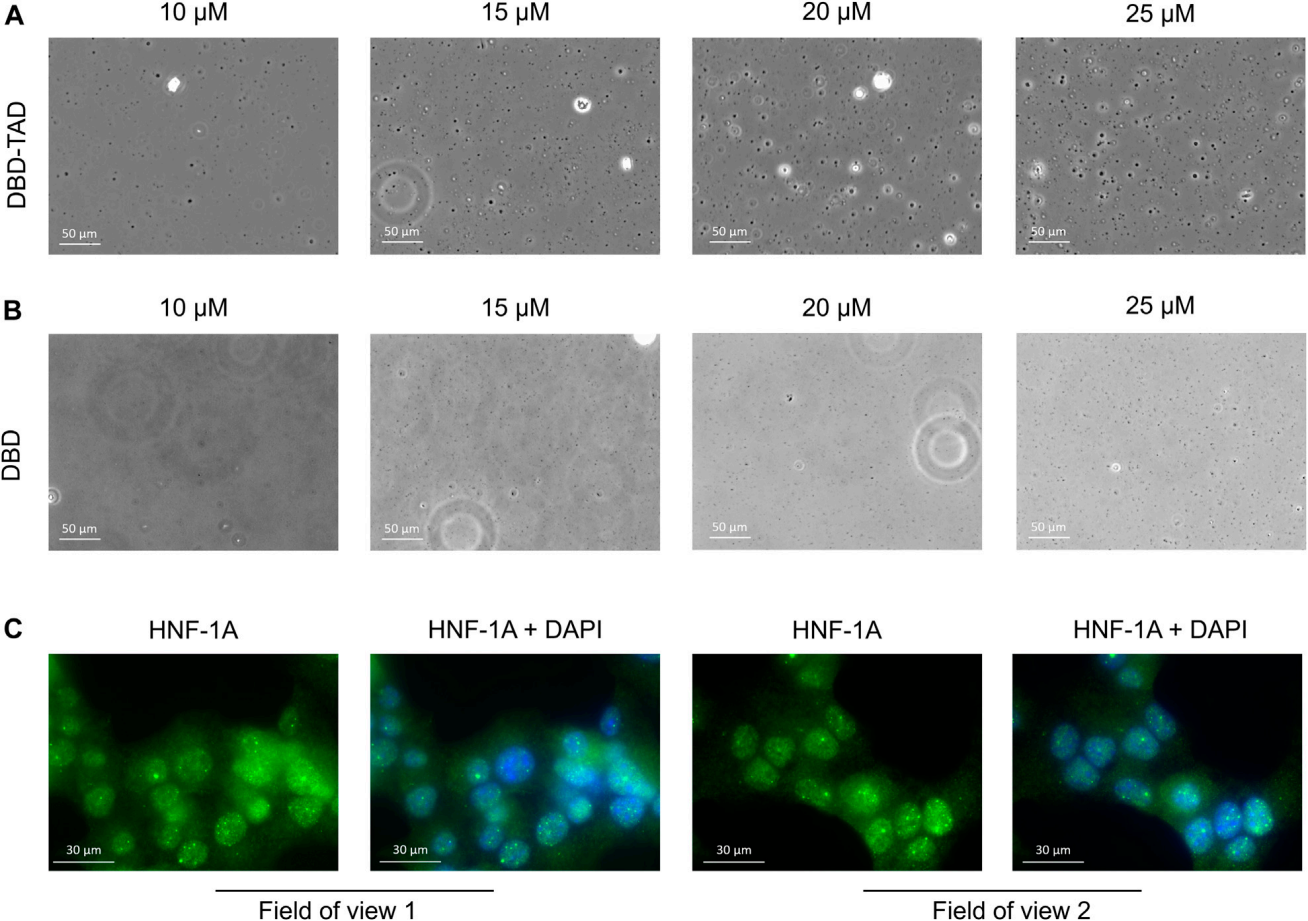
Initial evidence for LLPS behavior of HNF-1A **(A,B)**. Representative DIC microscopy images of purified DBD-TAD **(A)** or DBD **(B)** at different concentrations (10–25 µM) in the presence of 10% PEG8000 as a molecular crowding agent **(C)**. IF images of MIN6 β-like cells, stained for HNF-1A using an HNF-1A specific antibody and DNA using DAPI stain. Two fields of view from the same microscopy slide are presented, where the left image represents HNF-1A signals (green) and the right image a superposition of the same HNF-1A signal (green) and nuclear DAPI signal (blue).

Based on the FuzDrop predictions (Figure 1D), we suspected that the disordered TAD may have the potential to promote LLPS of HNF-1A. We observed that the purified DBD-TAD protein could not be concentrated above ca. 2.5 mg/mL. The protein solution increased in viscosity at higher concentrations, which may be due to the predicted formation of protein droplets, hydrogel or fibrous aggregates (Figure 1D) (Vendruscolo and Fuxreiter, 2022; Wang et al., 2022). To investigate the potential cause, we performed DIC microscopy using purified DBD-TAD and DBD (Figures 3A, B). As LLPS is a concentration-dependent process and HNF-1A condensation in the cell was expected to be modulated by the dense molecular environment of the nucleus, we used the crowding agent PEG8000 to mimic the native environment *in vitro*. We observed droplet formation for the DBD-TAD construct, with a concentration-dependent increase in droplet number (Figure 3A). Most droplets had a round shape, suggesting that they were liquid condensates, as opposed to aggregates. The DBD protein also produced droplets; however, higher protein concentrations were required, and the droplet size differed compared to the TAD-containing protein (Figure 3B). The latter indicates that the TAD may be a major driver of the LLPS behavior of HNF-1A. However, it is important to note that the comparison between the two proteins and their contribution to LLPS at a given molar concentration is not straightforward due to their different molecular size. Based on the FuzDrop prediction (Figure 1D), it may be possible that certain regions of the DBD contribute to LLPS of HNF-1A, such as residues 179–200 in the intrinsically disordered POU_S_-POU_H_ linker (Kind et al., 2022). Additional experimental evidence for the presence of HNF-1A-containing droplets *in vitro* may be acquired by performing fluorescence microscopy with a fluorescently labeled (DBD-) TAD, observing the mixing dynamics and potential fusion events between the droplets. The characteristic liquid-like behavior of biomolecular condensates could be tested using fluorescence recovery after photobleaching (FRAP) microscopy (Alberti et al., 2019; Ganser and Myong, 2020).

As transcription factors are normally present in the nM concentration range (copy numbers between 10^3^-10^6^ per mammalian cell) (Biggin, 2011), our experimental conditions do not fully recapitulate the protein concentration at endogenous levels. However, even with lower HNF-1A concentrations in the cell, phase separation may take place in the presence of other IDR-containing proteins promoting multivalent interactions. LLPS-dependent cellular compartments typically concentrate ten to several hundred different proteins (and often RNA molecules), whereby many of them contribute to the formation of the biomolecular condensate (Banani et al., 2017). We next investigated whether the suspected LLPS behavior of HNF-1A may lead to the formation of biological condensates in cells. We employed fluorescence microscopy to image the localization of endogenous full-length HNF-1A in fixed MIN6 β-like cells, revealing that HNF-1A was predominantly located in the nucleus (Figure 3C). Moreover, HNF-1A produced strong fluorescent punctae, which co-localized with the nuclear stain and may thus represent nuclear condensates (Figure 3C). It is important to note that the cell fixation using paraformaldehyde can significantly alter the appearance or presence of biomolecular condensates, which is why the presented results should be interpreted with care and verified with alternative methods (Irgen-Gioro et al., 2022). Live-cell imaging techniques will be valuable for verifying the existence of HNF-1A-containing nuclear condensates in pancreatic β-cells. The HNF-1A signal in living cells may be observed over the course of time to investigate their potential dynamic behavior in different cellular states. In addition, liquid-like properties of the putative condensates may be probed by FRAP microscopy (Alberti et al., 2019). In order to prevent artefacts due to overexpression of exogenous HNF-1A, the cells may be genetically engineered to endogenously express fluorescently labeled HNF-1A. In this case, a cell line expressing the fluorescent protein alone will serve as an important control (Alberti et al., 2019).

Following the verification of HNF-1A condensate formation, the biological function of the structures may be determined by the identification of other condensate components. HNF-1A may phase separate with other proteins, DNA, or RNA (Banani et al., 2017). Studies of HNF-1A DNA interactions may be initiated by an antibody-based identification of HNF-1A bound DNA fragments, such as in chromatin immuno-precipitation (ChIP) or the Cleavage Under Targets & Release Using Nuclease (CUT&RUN) technique (Park, 2009; Skene and Henikoff, 2017). The detected DNA fragments can point towards genomic locations, at which HNF-1A condensates nucleate and initiate gene transcription. Proteins interacting with HNF-1A may be identified using proteomic approaches (Roux et al., 2018; DeCaprio and Kohl, 2020), either investigating stable interaction partners by immuno-precipitation (IP) or assessing transient interactions by performing biotin-labelling of interaction partners based on proximity in the cellular environment (BioID). Co-localization studies with general components of the transcriptional machinery (e.g., RNA polymerase II, TFIID/H, Mediator, DSIF) could elucidate whether HNF-1A partakes in transcriptional condensates (Schier and Taatjes, 2020). This approach is exemplified by a study from Boija et al. (2018), which demonstrated that the transcription factors OCT4, GCN4, and ER recruit Mediator into phase-separated droplets *in vitro* and in cells and that their ability to do so is linked to gene activation potential.

### 3.7 TAD undergoes LLPS in molecular dynamics simulations

**FIGURE 4:**
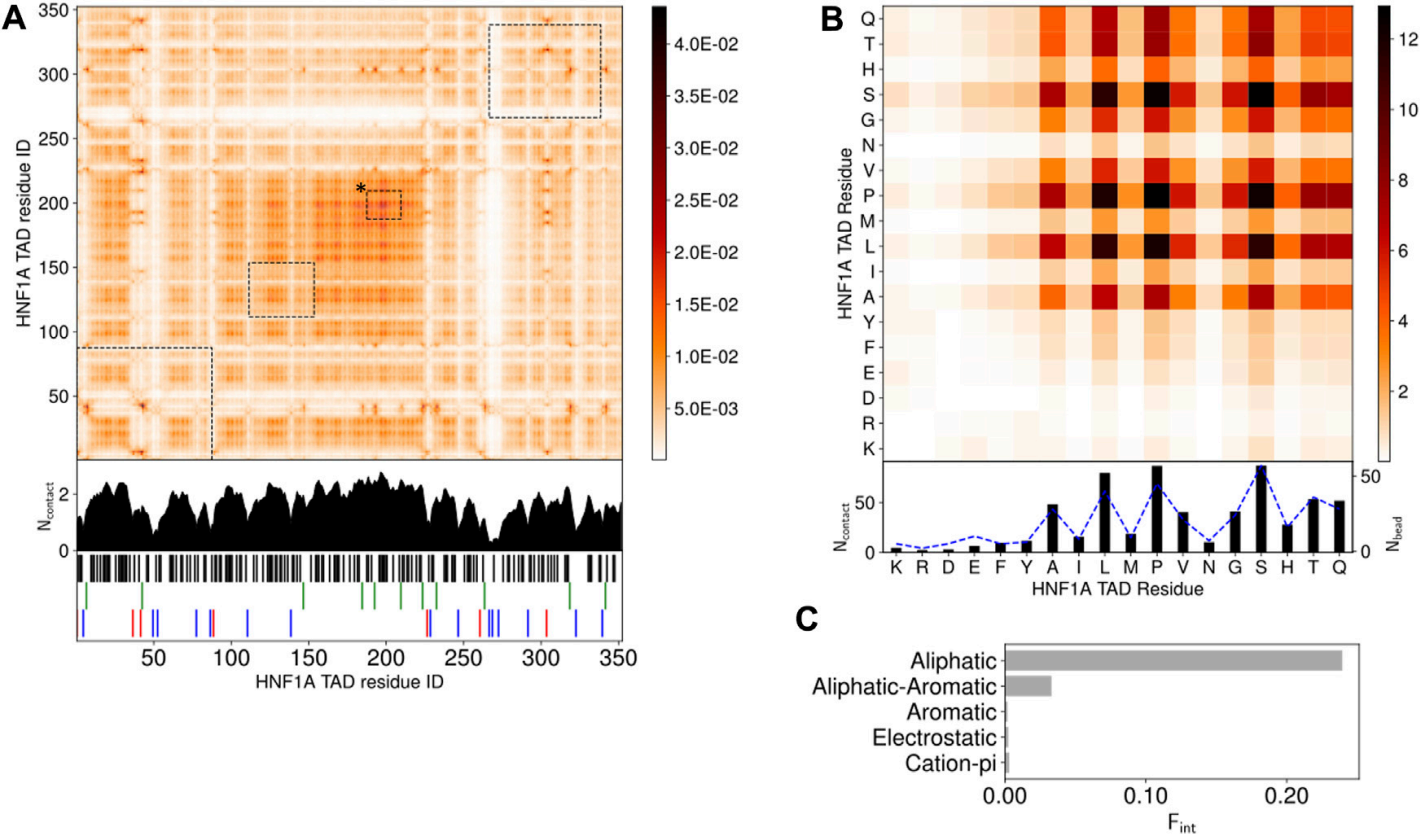
LLPS promoting TAD interactions, studied by droplet simulations **(A)**. Intermolecular contact map by residue index for 120 HNF-1A TAD molecules at 150 mM ion concentration and 300 K. The contacts in the contact maps by residue index are averaged in time (600 frames) and normalized by the number of HNF-1A TAD molecules (120) in the simulation. The 1D summation is shown below the contact map for each residue. Residues are categorized into 5 groups: cations (R, K)- red, anions (D, E)- blue, aromatic (F, Y, W)- green, aliphatic (A, C, I, L, M, P, V)- black, hydrophilic (G, N, S, H, Q, T)- white. The interactions between droplet promoting regions predicted by FuzDrop (Figure 1D) are shown by black dashed boxes. Box marked with “*” corresponds to FuzDrop DPR 466–488 **(B)**. Intermolecular contact map by residue type for 120 HNF-1A TAD molecules. The contact map is a matrix reduction of the contact map by residue index in **(A)**. The abundance for the residues, N_bead_, are shown by blue dashed lines **(C)**. Interaction summary for a droplet simulation with 120 HNF-1A TAD molecules at 150 mM ion concentration and 300 K. The fraction of interactions, F_int_, are aggregated by type and normalized by the total number of interactions.

To understand the LLPS propensity of the HNF-1A TAD, we undertook computational droplet formation simulations (Figure 4; Supplementary Figure S3; Supplementary Video S1). We assessed the internal structure of the droplets by using the 1 bead per amino acid (1BPA) molecular dynamics model (Driver et al., 2022), developed for the study of intrinsically disordered proteins in the nuclear pore complex (Ghavami et al., 2013; Ghavami et al., 2014). Self-assembly and clustering of individual monomers into phase-separated condensates can be a slow process to observe. To speed up this process, a condensed phase droplet is formed at the start of the simulation, which is then inserted into an empty dilute phase. If LLPS is favored, the droplet structure should remain stable throughout the subsequent simulation; if non-favored, the droplet would break up into a dilute phase of monomers.

We simulated the behavior of 120 HNF-1A TAD molecules and observed LLPS (Supplementary Video S1). In the beginning of the simulation, two to three small clusters with one to six HNF-1A TAD molecules co-existed with a large cluster containing approximately 115 HNF-1A TAD molecules. The smaller clusters rapidly fused with the big cluster, yielding a droplet of 120 HNF-1A TAD molecules that remained stably associated throughout the simulation period (Supplementary Video S1). By analyzing the interactions between the TAD molecules in detail, we found that intermolecular interactions along the whole length of the TAD contribute to LLPS (Figure 4A). The LLPS behavior of the TAD is driven by hydrophobic aliphatic-aliphatic contacts, with minimal aliphatic-aromatic interactions due to the low abundance of aromatic residues (Figure 4C). This is driven by contacts between Ala, Leu, Pro, and Val (Figure 4B). We also observed many interactions between hydrophilic residues (Gly, Ser, His, Thr, and Gln) driven by their high abundance (Figure 4B), but whose effect is reduced when interactions are normalized by amino acid abundance (Supplementary Figure S3). The predicted LLPS-promoting regions (Figure 1D) are highlighted on the contact map in Figure 4A. These regions partially align with the regions we found to form the most contacts, with only the FuzDrop DPR 466–488 showing full alignment (box marked with “*” in Figure 4A). The more extensive interactions discovered by the simulations indicate that the even distribution of aliphatic residues throughout the TAD contributes to LLPS, whereas an absence of intermolecular interactions among charged and polar residues, which make up 90% of the TAD, was evident (white stripes in Figure 4A). In summary, the results of our simulations corroborate with our *in vitro* data (Figure 3A) and suggest that several residues along the entire TAD collectively contribute to the droplet formation.

## 4 Conclusion

**FIGURE 5:**
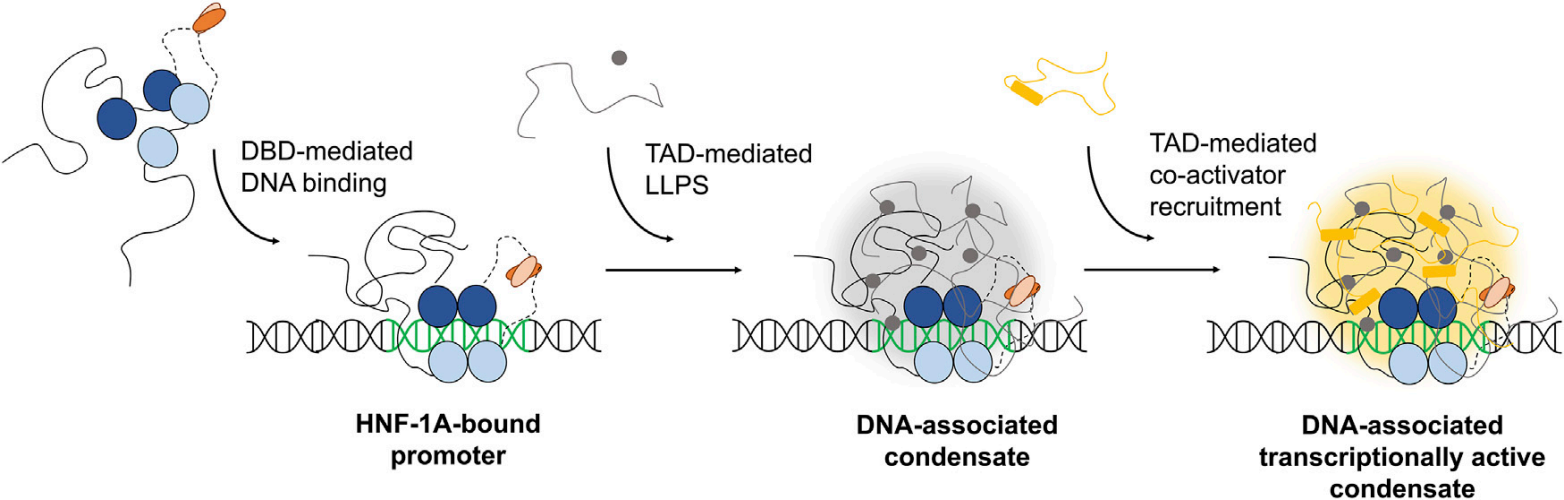
Hypothesis on the possible mechanism of TAD-dependent gene transcriptional activation by HNF-1A. This illustration is a highly simplified model with a focus on HNF-1A. Numerous other currently unknown molecules are likely to participate in the potential formation of transcriptional condensates at HNF-1A target sites. Blue: DBD of HNF-1A. Orange: DD of HNF-1A. Black line: TAD of HNF-1A. Grey: co-condensating molecules, e.g., proteins or RNA. Yellow: co-condensating co-activators.

Our study provides insights into the sequence features and structural properties of the uncharacterized TAD of HNF-1A. We developed the first purification protocol for a TAD-containing HNF-1A construct, which allowed us to study the TAD *in vitro*. We found that the TAD is intrinsically disordered, which may be crucial for the dynamic interaction with other proteins involved in gene transcriptional control. While we reproducibly obtained pure DBD-TAD samples, we observed a tendency for protein degradation from the C-terminal end of the TAD. Further efforts in construct design and protein purification may thus be undertaken in the future to improve the yield and protein stability. With a stable protein at hand, the use of NMR spectroscopy may provide insights into the structural features and interactions of the TAD at atomic resolution. Our functional data on the potential LLPS behavior of HNF-1A agreed with predictions and *in silico* simulations and supports the hypothesis that the TAD may drive the formation of biological condensates in cells. The reported LLPS of HNF-1A can potentially open a new avenue in β-cell research. The ability of HNF-1A to form biological condensates and the potential of the TAD to mediate protein-protein interactions may help to recruit specific co-activators to promoter sites of specific target genes, leading to transcriptionally active condensates (Figure 5). As a consequence, these nuclear assemblies may promote the initiation of essential gene transcriptional programs, allowing the β-cell to respond to external signals, e.g., changes in nutrient levels. Our study provides directions for future research on the biomolecular basis of HNF-1A function. An enhanced understanding of HNF-1A-mediated gene transcription and the variant-induced dysregulation thereof may potentially expose novel treatment targets in patients with HNF1A-MODY.

## Data Availability

The datasets presented in this study can be found in online repositories. The names of the repository/repositories and accession number(s) can be found in the article/Supplementary Material.

## References

[B1] Alberti S. Dormann D. (2019). Liquid-liquid phase separation in disease. Annu. Rev. Genet. 53, 171–194. 10.1146/annurev-genet-112618-043527 31430179

[B2] Alberti S. Gladfelter A. Mittag T. (2019). Considerations and challenges in studying liquid-liquid phase separation and biomolecular condensates. Cell 176 (3), 419–434. 10.1016/j.cell.2018.12.035 30682370 PMC6445271

[B3] Arai M. Ferreon J. C. Wright P. E. (2012). Quantitative analysis of multisite protein-ligand interactions by NMR: binding of intrinsically disordered p53 transactivation subdomains with the TAZ2 domain of CBP. J. Am. Chem. Soc. 134 (8), 3792–3803. 10.1021/ja209936u 22280219 PMC3290704

[B4] Bach I. Yaniv M. (1993). More potent transcriptional activators or a transdominant inhibitor of the HNF1 homeoprotein family are generated by alternative RNA processing. EMBO J. 12 (11), 4229–4242. 10.1002/j.1460-2075.1993.tb06107.x 7900999 PMC413717

[B5] Banani S. F. Lee H. O. Hyman A. A. Rosen M. K. (2017). Biomolecular condensates: organizers of cellular biochemistry. Nat. Rev. Mol. Cell Biol. 18 (5), 285–298. 10.1038/nrm.2017.7 28225081 PMC7434221

[B6] Bernadó P. Blackledge M. (2009). A self-consistent description of the conformational behavior of chemically denatured proteins from NMR and small angle scattering. Biophys. J. 97 (10), 2839–2845. 10.1016/j.bpj.2009.08.044 19917239 PMC2776250

[B7] Bernadó P. Mylonas E. Petoukhov M. V. Blackledge M. Svergun D. I. (2007). Structural characterization of flexible proteins using small-angle X-ray scattering. J. Am. Chem. Soc. 129 (17), 5656–5664. 10.1021/ja069124n 17411046

[B8] Biggin M. D. (2011). Animal transcription networks as highly connected, quantitative continua. Dev. Cell 21 (4), 611–626. 10.1016/j.devcel.2011.09.008 22014521

[B10] Bjørkhaug L. Bratland A. Njølstad P. R. Molven A. (2005). Functional dissection of the HNF-1alpha transcription factor: a study on nuclear localization and transcriptional activation. DNA Cell Biol. 24 (11), 661–669. 10.1089/dna.2005.24.661 16274290

[B11] Bjørkhaug L. Sagen J. V. Thorsby P. Søvik O. Molven A. Njølstad P. R. (2003). Hepatocyte nuclear factor-1 alpha gene mutations and diabetes in Norway. J. Clin. Endocrinol. Metab. 88 (2), 920–931. 10.1210/jc.2002-020945 12574234

[B12] Boija A. Klein I. A. Sabari B. R. Dall'Agnese A. Coffey E. L. Zamudio A. V. (2018). Transcription factors activate genes through the phase-separation capacity of their activation domains. Cell 175 (7), 1842–1855. 10.1016/j.cell.2018.10.042 30449618 PMC6295254

[B13] Bugge K. Brakti I. Fernandes C. B. Dreier J. E. Lundsgaard J. E. Olsen J. G. (2020). Interactions by disorder - a matter of context. Front. Mol. Biosci. 7, 110. 10.3389/fmolb.2020.00110 32613009 PMC7308724

[B14] Butt T. R. Edavettal S. C. Hall J. P. Mattern M. R. (2005). SUMO fusion technology for difficult-to-express proteins. Protein Expr. Purif. 43 (1), 1–9. 10.1016/j.pep.2005.03.016 16084395 PMC7129290

[B15] Calmettes P. Durand D. Desmadril M. Minard P. Receveur V. Smith J. C. (1994). How random is a highly denatured protein? Biophys. Chem. 53 (1-2), 105–113. 10.1016/0301-4622(94)00081-6 17020841

[B16] Chen C. Agnes F. Gelinas C. (1999). Mapping of a serine-rich domain essential for the transcriptional, antiapoptotic, and transforming activities of the v-Rel oncoprotein. Mol. Cell Biol. 19 (1), 307–316. 10.1128/MCB.19.1.307 9858554 PMC83888

[B17] Chi Y. I. Frantz J. D. Oh B. C. Hansen L. Dhe-Paganon S. Shoelson S. E. (2002). Diabetes mutations delineate an atypical POU domain in HNF-1alpha. Mol. Cell 10 (5), 1129–1137. 10.1016/s1097-2765(02)00704-9 12453420

[B18] Costa S. Almeida A. Castro A. Domingues L. (2014). Fusion tags for protein solubility, purification and immunogenicity in *Escherichia coli*: the novel Fh8 system. Front. Microbiol. 5, 63. 10.3389/fmicb.2014.00063 24600443 PMC3928792

[B19] Day E. K. Sosale N. G. Lazzara M. J. (2016). Cell signaling regulation by protein phosphorylation: a multivariate, heterogeneous, and context-dependent process. Curr. Opin. Biotechnol. 40, 185–192. 10.1016/j.copbio.2016.06.005 27393828 PMC4975652

[B20] De Francesco R. Pastore A. Vecchio G. Cortese R. (1991). Circular dichroism study on the conformational stability of the dimerization domain of transcription factor LFB1. Biochemistry 30 (1), 143–147. 10.1021/bi00215a021 1988015

[B21] DeCaprio J. Kohl T. O. (2020). Immunoprecipitation. Cold Spring Harb. Protoc. 2020 (11), pdb.top098509. 10.1101/pdb.top098509 29196601

[B83] DeLano W. L. (2002). PyMOL: An Open-Source Molecular Graphics Tool. CCP4 Newsletter 40:11.

[B22] DelRosso N. Tycko J. Suzuki P. Andrews C. Aradhana M. Mukund A. (2023). Large-scale mapping and mutagenesis of human transcriptional effector domains. Nature 616 (7956), 365–372. 10.1038/s41586-023-05906-y 37020022 PMC10484233

[B23] Dignon G. L. Best R. B. Mittal J. (2020). Biomolecular phase separation: from molecular driving forces to macroscopic properties. Annu. Rev. Phys. Chem. 71, 53–75. 10.1146/annurev-physchem-071819-113553 32312191 PMC7469089

[B24] Driver M. Postema J. Onck P. R. (2022). Complementary or competing interactions?: effects of DPRs and RNA on FUS condensates, and their implications in ALS progression. Biophysical J. 121, 472. 10.1016/j.bpj.2021.11.414

[B25] Durand D. Vives C. Cannella D. Perez J. Pebay-Peyroula E. Vachette P. (2010). NADPH oxidase activator p67(phox) behaves in solution as a multidomain protein with semi-flexible linkers. J. Struct. Biol. 169 (1), 45–53. 10.1016/j.jsb.2009.08.009 19723583

[B26] Dyson H. J. (2013). “Coupled folding and binding,” in Encyclopedia of Biophysics. Editor Roberts G. C. K. 10.1007/978-3-642-16712-6_174

[B27] Dyson H. J. Wright P. E. (2005). Intrinsically unstructured proteins and their functions. Nat. Rev. Mol. Cell Biol. 6 (3), 197–208. 10.1038/nrm1589 15738986

[B28] Erdős G. Pajkos M. Dosztányi Z. (2021). IUPred3: prediction of protein disorder enhanced with unambiguous experimental annotation and visualization of evolutionary conservation. Nucleic Acids Res. 49 (1), W297–W303. 10.1093/nar/gkab408 34048569 PMC8262696

[B29] Fitzkee N. C. Rose G. D. (2004). Reassessing random-coil statistics in unfolded proteins. Proc. Natl. Acad. Sci. U. S. A. 101 (34), 12497–12502. 10.1073/pnas.0404236101 15314216 PMC514656

[B30] Flannick J. Johansson S. Njølstad P. R. (2016). Common and rare forms of diabetes mellitus: towards a continuum of diabetes subtypes. Nat. Rev. Endocrinol. 12 (7), 394–406. 10.1038/nrendo.2016.50 27080136

[B31] Frietze S. Farnham P. J. (2011). Transcription factor effector domains. Subcell. Biochem. 52, 261–277. 10.1007/978-90-481-9069-0_12 21557087 PMC4151296

[B32] Ganser L. R. Myong S. (2020). Methods to study phase-separated condensates and the underlying molecular interactions. Trends Biochem. Sci. 45 (11), 1004–1005. 10.1016/j.tibs.2020.05.011 32561165 PMC7697221

[B33] Ghavami A. van der Giessen E. Onck P. R. (2013). Coarse-Grained potentials for local interactions in unfolded proteins. J. Chem. Theory Comput. 9 (1), 432–440. 10.1021/ct300684j 26589045

[B34] Ghavami A. Veenhoff L. M. van der Giessen E. Onck P. R. (2014). Probing the disordered domain of the nuclear pore complex through coarse-grained molecular dynamics simulations. Biophys. J. 107 (6), 1393–1402. 10.1016/j.bpj.2014.07.060 25229147 PMC4167297

[B35] Hammarström M. Woestenenk E. A. Hellgren N. Härd T. Berglund H. (2006). Effect of N-terminal solubility enhancing fusion proteins on yield of purified target protein. J. Struct. Funct. Genomics 7 (1), 1–14. 10.1007/s10969-005-9003-7 16850178

[B36] Hatos A. Tosatto S. C. E. Vendruscolo M. Fuxreiter M. (2022). FuzDrop on AlphaFold: visualizing the sequence-dependent propensity of liquid-liquid phase separation and aggregation of proteins. Nucleic Acids Res. 50 (1), W337–W344. 10.1093/nar/gkac386 35610022 PMC9252777

[B37] Hunter T. (2007). The age of crosstalk: phosphorylation, ubiquitination, and beyond. Mol. Cell 28 (5), 730–738. 10.1016/j.molcel.2007.11.019 18082598

[B38] Irgen-Gioro S. Yoshida S. Walling V. Chong S. (2022). Fixation can change the appearance of phase separation in living cells. Elife 11, e79903. 10.7554/eLife.79903 36444977 PMC9817179

[B39] Jumper J. Evans R. Pritzel A. Green T. Figurnov M. Ronneberger O. (2021). Highly accurate protein structure prediction with AlphaFold. Nature 596 (7873), 583–589. 10.1038/s41586-021-03819-2 34265844 PMC8371605

[B40] Kahnt M. Klementiev K. Haghighat V. Weninger C. Plivelic T. S. Terry A. E. (2021). Measurement of the coherent beam properties at the CoSAXS beamline. J. Synchrotron Radiat. 28 (6), 1948–1953. 10.1107/S1600577521009140 34738950 PMC8570205

[B41] Katzen F. (2007). Gateway(®) recombinational cloning: a biological operating system. Expert Opin. Drug Discov. 2 (4), 571–589. 10.1517/17460441.2.4.571 23484762

[B42] Kim D. H. Wright A. Han K. H. (2017). An NMR study on the intrinsically disordered core transactivation domain of human glucocorticoid receptor. BMB Rep. 50 (10), 522–527. 10.5483/bmbrep.2017.50.10.152 28946939 PMC5683822

[B43] Kind L. Raasakka A. Molnes J. Aukrust I. Bjørkhaug L. Njølstad P. R. (2022). Structural and biophysical characterization of transcription factor HNF-1A as a tool to study MODY3 diabetes variants. J. Biol. Chem. 298 (4), 101803. 10.1016/j.jbc.2022.101803 35257744 PMC8988010

[B44] Krentz N. A. J. Gloyn A. L. (2020). Insights into pancreatic islet cell dysfunction from type 2 diabetes mellitus genetics. Nat. Rev. Endocrinol. 16 (4), 202–212. 10.1038/s41574-020-0325-0 32099086

[B45] Krois A. S. Dyson H. J. Wright P. E. (2018). Long-range regulation of p53 DNA binding by its intrinsically disordered N-terminal transactivation domain. Proc. Natl. Acad. Sci. U. S. A. 115 (48), E11302–E11310. 10.1073/pnas.1814051115 30420502 PMC6275486

[B46] Kumar M. Michael S. Alvarado-Valverde J. Meszaros B. Samano-Sanchez H. Zeke A. (2022). The eukaryotic linear motif resource: 2022 release. Nucleic Acids Res. 50 (1), D497–D508. 10.1093/nar/gkab975 34718738 PMC8728146

[B47] Leibly D. J. Nguyen T. N. Kao L. T. Hewitt S. N. Barrett L. K. Van Voorhis W. C. (2012). Stabilizing additives added during cell lysis aid in the solubilization of recombinant proteins. PLoS One 7 (12), e52482. 10.1371/journal.pone.0052482 23285060 PMC3527557

[B48] Liu J. Perumal N. B. Oldfield C. J. Su E. W. Uversky V. N. Dunker A. K. (2006). Intrinsic disorder in transcription factors. Biochemistry 45 (22), 6873–6888. 10.1021/bi0602718 16734424 PMC2538555

[B49] Madeira F. Pearce M. Tivey A. R. N. Basutkar P. Lee J. Edbali O. (2022). Search and sequence analysis tools services from EMBL-EBI in 2022. Nucleic Acids Res. 50, W276–W279. 10.1093/nar/gkac240 35412617 PMC9252731

[B50] Manalastas-Cantos K. Konarev P. V. Hajizadeh N. R. Kikhney A. G. Petoukhov M. V. Molodenskiy D. S. (2021). ATSAS 3.0: expanded functionality and new tools for small-angle scattering data analysis. J. Appl. Crystallogr. 54 (1), 343–355. 10.1107/S1600576720013412 33833657 PMC7941305

[B51] Mar M. Nitsenko K. Heidarsson P. O. (2023). Multifunctional intrinsically disordered regions in transcription factors. Chemistry 29 (21), e202203369. 10.1002/chem.202203369 36648282

[B52] Mathieu C. Pappu R. V. Taylor J. P. (2020). Beyond aggregation: pathological phase transitions in neurodegenerative disease. Science 370 (6512), 56–60. 10.1126/science.abb8032 33004511 PMC8359821

[B53] Mészáros B. Simon I. Dosztányi Z. (2009). Prediction of protein binding regions in disordered proteins. PLoS Comput. Biol. 5 (5), e1000376. 10.1371/journal.pcbi.1000376 19412530 PMC2671142

[B54] Micsonai A. Wien F. Bulyaki E. Kun J. Moussong E. Lee Y. H. (2018). BeStSel: a web server for accurate protein secondary structure prediction and fold recognition from the circular dichroism spectra. Nucleic Acids Res. 46 (1), W315–W322. 10.1093/nar/gky497 29893907 PMC6031044

[B55] Micsonai A. Wien F. Kernya L. Lee Y. H. Goto Y. Refregiers M. (2015). Accurate secondary structure prediction and fold recognition for circular dichroism spectroscopy. Proc. Natl. Acad. Sci. U. S. A. 112 (24), E3095–E3103. 10.1073/pnas.1500851112 26038575 PMC4475991

[B56] Miles A. J. Drew E. D. Wallace B. A. (2023). DichroIDP: a method for analyses of intrinsically disordered proteins using circular dichroism spectroscopy. Commun. Biol. 6 (1), 823. 10.1038/s42003-023-05178-2 37553525 PMC10409736

[B57] Miles A. J. Wallace B. A. (2018). CDtoolX, a downloadable software package for processing and analyses of circular dichroism spectroscopic data. Protein Sci. 27 (9), 1717–1722. 10.1002/pro.3474 30168221 PMC6194270

[B58] Mitchell P. J. Tjian R. (1989). Transcriptional regulation in mammalian cells by sequence-specific DNA binding proteins. Science 245 (4916), 371–378. 10.1126/science.2667136 2667136

[B59] Miyazaki J. Araki K. Yamato E. Ikegami H. Asano T. Shibasaki Y. (1990). Establishment of a pancreatic beta cell line that retains glucose-inducible insulin secretion: special reference to expression of glucose transporter isoforms. Endocrinology 127 (1), 126–132. 10.1210/endo-127-1-126 2163307

[B60] Naar A. M. Lemon B. D. Tjian R. (2001). Transcriptional coactivator complexes. Annu. Rev. Biochem. 70, 475–501. 10.1146/annurev.biochem.70.1.475 11395415

[B61] Najmi L. A. Aukrust I. Flannick J. Molnes J. Burtt N. Molven A. (2017). Functional investigations of HNF1A identify rare variants as risk factors for type 2 diabetes in the general population. Diabetes 66 (2), 335–346. 10.2337/db16-0460 27899486 PMC5860263

[B62] Narayana N. Hua Q. Weiss M. A. (2001). The dimerization domain of HNF-1alpha: structure and plasticity of an intertwined four-helix bundle with application to diabetes mellitus. J. Mol. Biol. 310 (3), 635–658. 10.1006/jmbi.2001.4780 11439029

[B63] Narayana N. Phillips N. B. Hua Q. X. Jia W. Weiss M. A. (2006). Diabetes mellitus due to misfolding of a beta-cell transcription factor: stereospecific frustration of a schellman motif in HNF-1alpha. J. Mol. Biol. 362 (3), 414–429. 10.1016/j.jmb.2006.06.086 16930618

[B64] O'Shea C. Staby L. Bendsen S. K. Tidemand F. G. Redsted A. Willemoes M. (2017). Structures and short linear motif of disordered transcription factor regions provide clues to the interactome of the cellular hub protein radical-induced cell Death1. J. Biol. Chem. 292 (2), 512–527. 10.1074/jbc.M116.753426 27881680 PMC5241728

[B65] Odom D. T. Zizlsperger N. Gordon D. B. Bell G. W. Rinaldi N. J. Murray H. L. (2004). Control of pancreas and liver gene expression by HNF transcription factors. Science 303 (5662), 1378–1381. 10.1126/science.1089769 14988562 PMC3012624

[B66] Oldfield C. J. Meng J. Yang J. Y. Yang M. Q. Uversky V. N. Dunker A. K. (2008). Flexible nets: disorder and induced fit in the associations of p53 and 14-3-3 with their partners. BMC Genomics 9 (1), S1. 10.1186/1471-2164-9-S1-S1 PMC2386051 18366598

[B67] Park P. J. (2009). ChIP-seq: advantages and challenges of a maturing technology. Nat. Rev. Genet. 10 (10), 669–680. 10.1038/nrg2641 19736561 PMC3191340

[B68] Piiadov V. Ares de Araujo E. Oliveira Neto M. Craievich A. F. Polikarpov I. (2019). SAXSMoW 2.0: online calculator of the molecular weight of proteins in dilute solution from experimental SAXS data measured on a relative scale. Protein Sci. 28 (2), 454–463. 10.1002/pro.3528 30371978 PMC6319763

[B69] Piovesan D. Walsh I. Minervini G. Tosatto S. C. E. (2017). FELLS: fast estimator of latent local structure. Bioinformatics 33 (12), 1889–1891. 10.1093/bioinformatics/btx085 28186245

[B70] Poitout V. Olson L. K. Robertson R. P. (1996). Insulin-secreting cell lines: classification, characteristics and potential applications. Diabetes Metab. 22 (1), 7–14. 8697299

[B71] Rey-Campos J. Chouard T. Yaniv M. Cereghini S. (1991). vHNF1 is a homeoprotein that activates transcription and forms heterodimers with HNF1. EMBO J. 10 (6), 1445–1457. 10.1002/j.1460-2075.1991.tb07665.x 1673926 PMC452807

[B72] Raasakka A. Linxweiler H. Brophy P. J. Sherman D. L. Kursula P. (2019). Direct binding of the flexible C-terminal segment of Periaxin to β4 Integrin suggests a molecular basis for CMT4F. Front. Mol. Neurosci. 12, 84. 10.3389/fnmol.2019.00084 31024253 PMC6465933

[B73] Ramalli S. G. Miles A. J. Janes R. W. Wallace B. A. (2022). The PCDDB (protein circular dichroism data bank): A bioinformatics resource for protein characterisations and methods development. J. Mol. Biol. 434 (11), 167441. 10.1016/j.jmb.2022.167441 34999124

[B74] Rambo R. P. Tainer J. A. (2013). Accurate assessment of mass, models and resolution by small-angle scattering. Nature 496 (7446), 477–481. 10.1038/nature12070 23619693 PMC3714217

[B75] Riedl T. Egly J. M. (2000). Phosphorylation in transcription: the CTD and more. Gene Expr. 9 (1-2), 3–13. 10.3727/000000001783992704 11097421 PMC5964956

[B76] Robert X. Gouet P. (2014). Deciphering key features in protein structures with the new ENDscript server. Nucleic Acids Res. 42, W320–W324. 10.1093/nar/gku316 24753421 PMC4086106

[B77] Roux K. J. Kim D. I. Burke B. May D. G. (2018). BioID: A screen for protein-protein interactions. Curr. Protoc. Protein Sci. 91, 11–19. 10.1002/cpps.51 PMC6028010 29516480

[B78] Ryu J. Park H. H. Park J. H. Lee H. J. Rhee W. J. Park T. H. (2016). Soluble expression and stability enhancement of transcription factors using 30Kc19 cell-penetrating protein. Appl. Microbiol. Biotechnol. 100 (8), 3523–3532. 10.1007/s00253-015-7199-4 26668030

[B79] Sabari B. R. (2020). Biomolecular condensates and gene activation in development and disease. Dev. Cell 55 (1), 84–96. 10.1016/j.devcel.2020.09.005 33049213

[B80] Sanborn A. L. Yeh B. T. Feigerle J. T. Hao C. V. Townshend R. J. Lieberman Aiden E. (2021). Simple biochemical features underlie transcriptional activation domain diversity and dynamic, fuzzy binding to Mediator. Elife 10, e68068. 10.7554/eLife.68068 33904398 PMC8137143

[B81] Schier A. C. Taatjes D. J. (2020). Structure and mechanism of the RNA polymerase II transcription machinery. Genes Dev. 34 (7-8), 465–488. 10.1101/gad.335679.119 32238450 PMC7111264

[B82] Schindelin J. Arganda-Carreras I. Frise E. Kaynig V. Longair M. Pietzsch T. (2012). Fiji: an open-source platform for biological-image analysis. Nat. Methods 9 (7), 676–682. 10.1038/nmeth.2019 22743772 PMC3855844

[B84] Servitja J. M. Ferrer J. (2004). Transcriptional networks controlling pancreatic development and beta cell function. Diabetologia 47 (4), 597–613. 10.1007/s00125-004-1368-9 15298336

[B85] Servitja J. M. Pignatelli M. Maestro M. A. Cardalda C. Boj S. F. Lozano J. (2009). Hnf1alpha (MODY3) controls tissue-specific transcriptional programs and exerts opposed effects on cell growth in pancreatic islets and liver. Mol. Cell Biol. 29 (11), 2945–2959. 10.1128/MCB.01389-08 19289501 PMC2682018

[B86] Sievers F. Higgins D. G. (2021). The clustal Omega multiple alignment package. Methods Mol. Biol. 2231, 3–16. 10.1007/978-1-0716-1036-7_1 33289883

[B87] Skene P. J. Henikoff S. (2017). An efficient targeted nuclease strategy for high-resolution mapping of DNA binding sites. Elife 6, e21856. 10.7554/eLife.21856 28079019 PMC5310842

[B88] Sørensen H. P. Mortensen K. K. (2005). Soluble expression of recombinant proteins in the cytoplasm of *Escherichia coli* . Microb. Cell Fact. 4 (1), 1. 10.1186/1475-2859-4-1 15629064 PMC544838

[B89] Staby L. Due A. D. Kunze M. B. A. Jorgensen M. L. M. Skriver K. Kragelund B. B. (2021). Flanking disorder of the folded αα-hub domain from radical induced cell Death1 affects transcription factor binding by ensemble redistribution. J. Mol. Biol. 433 (24), 167320. 10.1016/j.jmb.2021.167320 34687712

[B90] Staby L. O'Shea C. Willemoes M. Theisen F. Kragelund B. B. Skriver K. (2017). Eukaryotic transcription factors: paradigms of protein intrinsic disorder. Biochem. J. 474 (15), 2509–2532. 10.1042/BCJ20160631 28701416

[B91] Staller M. V. Ramirez E. Kotha S. R. Holehouse A. S. Pappu R. V. Cohen B. A. (2022). Directed mutational scanning reveals a balance between acidic and hydrophobic residues in strong human activation domains. Cell Syst. 13 (4), 334–345.e5. 10.1016/j.cels.2022.01.002 35120642 PMC9241528

[B92] Straub S. G. Sharp G. W. (2002). Glucose-stimulated signaling pathways in biphasic insulin secretion. Diabetes Metab. Res. Rev. 18 (6), 451–463. 10.1002/dmrr.329 12469359

[B93] Tanaka M. Clouston W. M. Herr W. (1994). The Oct-2 glutamine-rich and proline-rich activation domains can synergize with each other or duplicates of themselves to activate transcription. Mol. Cell Biol. 14 (9), 6046–6055. 10.1128/mcb.14.9.6046 8065338 PMC359131

[B94] Toniatti C. Monaci P. Nicosia A. Cortese R. Ciliberto G. (1993). A bipartite activation domain is responsible for the activity of transcription factor HNF1/LFB1 in cells of hepatic and nonhepatic origin. DNA Cell Biol. 12 (3), 199–208. 10.1089/dna.1993.12.199 8466643

[B95] Tria G. Mertens H. D. Kachala M. Svergun D. I. (2015). Advanced ensemble modelling of flexible macromolecules using X-ray solution scattering. IUCrJ 2 (2), 207–217. 10.1107/S205225251500202X PMC4392415 25866658

[B96] UniProt C. (2021). UniProt: the universal protein knowledgebase in 2021. Nucleic Acids Res. 49 (1), D480–D489. 10.1093/nar/gkaa1100 33237286 PMC7778908

[B97] Uversky V. N. (2002). What does it mean to be natively unfolded? Eur. J. Biochem. 269 (1), 2–12. 10.1046/j.0014-2956.2001.02649.x 11784292

[B98] Valentini E. Kikhney A. G. Previtali G. Jeffries C. M. Svergun D. I. (2015). SASBDB, a repository for biological small-angle scattering data. Nucleic Acids Res. 43, D357–D363. 10.1093/nar/gku1047 25352555 PMC4383894

[B99] Varadi M. Anyango S. Deshpande M. Nair S. Natassia C. Yordanova G. (2022). AlphaFold protein structure database: massively expanding the structural coverage of protein-sequence space with high-accuracy models. Nucleic Acids Res. 50 (1), D439–D444. 10.1093/nar/gkab1061 34791371 PMC8728224

[B100] Vendruscolo M. Fuxreiter M. (2022). Sequence determinants of the aggregation of proteins within condensates generated by liquid-liquid phase separation. J. Mol. Biol. 434 (1), 167201. 10.1016/j.jmb.2021.167201 34391803

[B101] Wang H. Hagenfeldt-Johansson K. Otten L. A. Gauthier B. R. Herrera P. L. Wollheim C. B. (2002). Experimental models of transcription factor-associated maturity-onset diabetes of the young. Diabetes 51 (3), S333–S342. 10.2337/diabetes.51.2007.s333 12475772

[B102] Wang Z. Lou J. Zhang H. (2022). Essence determines phenomenon: assaying the material properties of biological condensates. J. Biol. Chem. 298 (4), 101782. 10.1016/j.jbc.2022.101782 35245500 PMC8958544

[B103] Zhang H. Colclough K. Gloyn A. L. Pollin T. I. (2021). Monogenic diabetes: a gateway to precision medicine in diabetes. J. Clin. Invest. 131 (3), e142244. 10.1172/JCI142244 33529164 PMC7843214

