# Supplementary material for: Structural properties of the HNF-1A transactivation domain
Source: Front Mol Biosci. 2023 Oct 16;10:1249939. doi: 10.3389/fmolb.2023.1249939 (PMC10613711; doi:10.3389/fmolb.2023.1249939)
Supplement: Supplementary file 1 [file DataSheet1.PDF]

Supplementary Material

Structural properties of the HNF-1A transactivation domain

Laura Kind*, Mark Driver, Arne Raasakka, Patrick R. Onck, Pål Rasmus Njølstad, Thomas Arnesen & Petri Kursula

*** Correspondence:** Laura Kind, laura.kind@uib.no

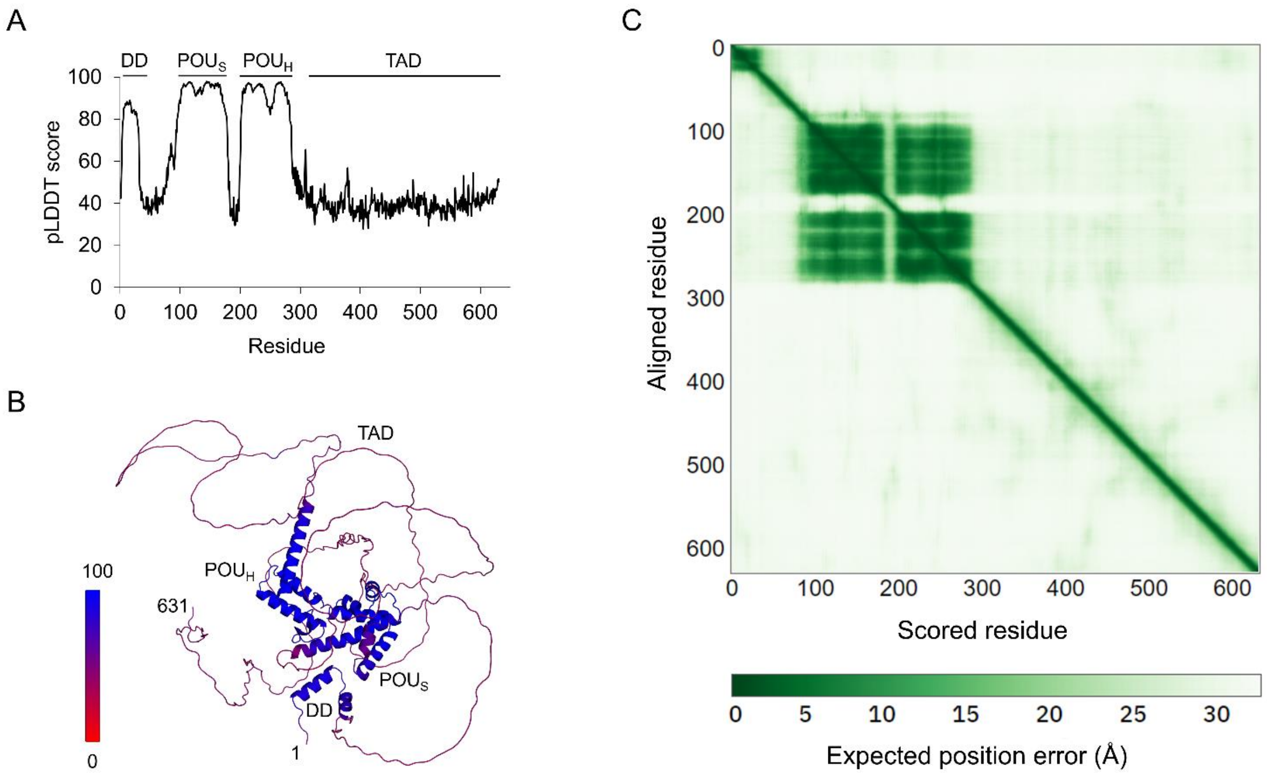

Fig. S1. Prediction scores for the tertiary structure model of *Hs*HNF-1A generated by AlphaFold. A. pLDDT score per HNF-1A residue. **B.** HNF-1A AlphaFold model (AF-P20823-F1-model_v4.pdb) colored according to the pLDDT score shown in A. **C.** Predicted aligned error plot for the same AlphaFold HNF-1A model.

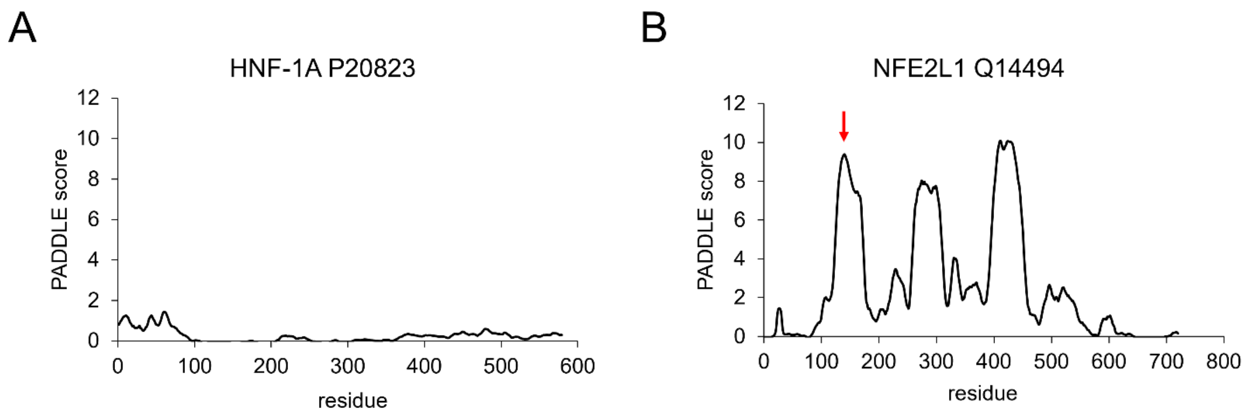

Fig. S2. Prediction of activation domains in HNF-1A using the "Predictor of Activation Domains using Deep Learning in Eukaryotes" (PADDLE) algorithm. A. PADDLE scores for HNF-1A (P20823). **B.** PADDLE scores for the reference transcription factor NFE2L1 (Q14494), for which one predicted activation domain (indicated with red arrow, centered around residue 140) was experimentally verified (Sanborn et al., 2021).

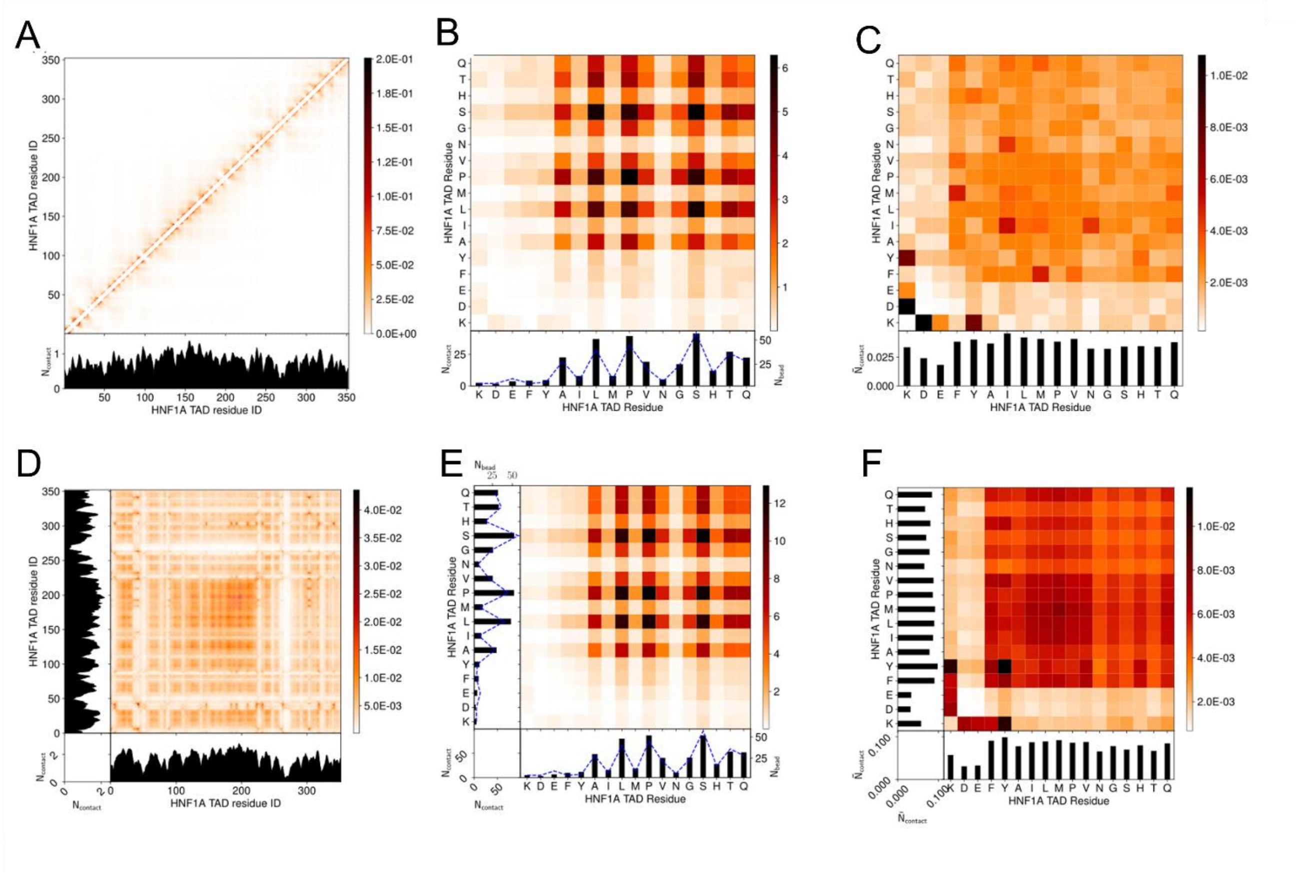

Figure S3: Single molecule contact maps for a droplet simulation with 120 HNF-1A TAD molecules at 150 mM ion concentration and 300 K. A. Intramolecular contact map by residue index. **B.** Intramolecular contact map by residue type. **C.** Intramolecular contact map by residue type, normalized by residue abundance. **D.** Intermolecular contact map by residue index. **E.** Intermolecular contact map by residue type. **F.** Intermolecular contact map by residue type, normalized by residue abundance. The contacts in the contact maps by residue index (left column) are normalized by the number of frames used (600) and the number of HNF1A TAD molecules (120). The contact maps aggregated by residue type (centre column) are a matrix reduction of the contact map by residue index (left column). The abundance for the residues, N_{bead}, are shown by blue dashed lines. The contact maps (right column) are normalized by the square root of the relative bead abundance in the molecules.

Table S1. Small-angle X-ray scattering parameters for HNF-1A DBD and DBD-TAD.

Protein	**DBD**	**DBD-TAD**
**Data collection**		
Instrument	P12 beamline, PETRA III, DESY, Hamburg, Germany	CoSAXS beamline, MAX IV Laboratory, Lund, Sweden
Wavelength (nm)	0.124	0.100
Angular range (nm^{-1})	0.03 – 7.4	0.0038 – 6.4
Temperature (°C)	10	10
SAXS mode	Batch	Batch
Exposure time (s)	0.045	0.020
Number of frames	39	300
Concentration (mg/ml)	2.6	2.5
Buffer	20 mM Hepes (pH 8.0), 500 mM NaCl, 1 mM TCEP	4 mM Tris pH 8.5, 100 mM NaCl, 1 mM TCEP
Method for scaling intensities	relative	Absolute scaling (cm^{-1}) referenced to water
Measurement type	mail-in	on-site
**Software**		
Primary data reduction and processing	*PRIMUSqt* in ATSAS 3.2.1 (Manalastas-Cantos et al., 2021)	
Data validation and analysis	*PRIMUSqt* in ATSAS 3.2.1 (Manalastas-Cantos et al., 2021)	
Ensemble modelling	*EOM* via *ATSAS* online (https://www.embl-hamburg.de/biosaxs/atsas-online/) (Bernado et al., 2007; Tria et al., 2015)	
Graphical representation	PyMOL	
**Structural parameters**		
Guinier analysis (Manalastas-Cantos et al., 2021)		
sR_g range	0.64 – 1.28	0.76 – 1.16
$I_0 \pm \sigma$	142.8 ± 0.8	0.039 ± 0.000
R_g (nm) ± σ	2.65 ± 0.02	4.85 ± 0.09
Linear fit assessment (*AUTORG* fidelity)	0.71	0.72
P(r) function (Manalastas-Cantos et al., 2021)		
s range ($Å^{-1}$)	8.0×10^{-3}–3.0×10^{-1}	9.2×10^{-3}–1.5×10^{-1}
$I_0 \pm \sigma$	145.4 ± 0.5	0.038 ± 0.000
R_g (nm) ± σ	2.83 ± 0.02	4.82 ± 0.03
D_{max} (nm)	10.2	17.0
P(r) reciprocal space fit: χ^2, CorMap p-value	1.01, 0.06	1.24, 0.01
Debye formalism (Calmettes et al., 1994; Fitzkee and Rose, 2004; Bernado and Blackledge, 2009; Raasakka et al., 2019)		
R_g (nm)	n.d.	5.1
**Molecular weight estimates**		
M (kDa), theoretical from sequence	22.8	62.2
M (kDa), from I_0	14.1<br>based on BSA as reference protein	54.0<br>based on absolute scale
M (kDa), Q_p, PRIMUSqt	15.1	82.7
M (kDa), MoW, PRIMUSqt	11.6	35.0
M (kDa), V_c, PRIMUSqt	16.0	52.8
M (kDa), Size and shape, PRIMUSqt	21.2	126.8
M (kDa), Bayesian Inference, PRIMUSqt;<br>M probability;<br>[Credibility interval];<br>interval probability	16.1;<br>54.5%;<br>[13.1; 17.8];<br>92.4%	58.2;<br>13.9%;<br>[31.3; 84,3];<br>91.3%
M (kDa), MoW 2.0 (Piiadov et al., 2019)	n.d.	58.2
**Ensemble modelling**		
Crystal structure used for rigid bodies	n.a.	1IC8 (Chi et al., 2002)

HNF-1A residues modelled as rigid bodies	n.a.	87 – 180 (fixed in space), 209 - 276
Symmetry	n.a.	P1
Parameters	n.a.	Default, constant subtraction allowed, native-like chain models, number of theoretical curves = 10 000
Number of representative structures	n.a.	7
R_g (nm), final ensemble	n.a.	6.397
D_{max} (nm), final ensemble	n.a.	19.84
R_{flex} (random)	n.a.	89.3% (86.7%)
R_σ	n.a.	1.71
χ^2	n.a.	1.099
**References**		
Publications including SAXS dataset	(Kind et al., 2022)	n.a.
SASBDB ID	SASDSZ8	SASDS29

References

The PyMOL Molecular Graphics System, Version 1.2r3pre, Schrödinger, LLC.

Bernado, P., and Blackledge, M. (2009). A self-consistent description of the conformational behavior of chemically denatured proteins from NMR and small angle scattering. *Biophys J* 97(10)**,** 2839-2845. doi: 10.1016/j.bpj.2009.08.044.

Bernado, P., Mylonas, E., Petoukhov, M.V., Blackledge, M., and Svergun, D.I. (2007). Structural characterization of flexible proteins using small-angle X-ray scattering. *J Am Chem Soc* 129(17)**,** 5656-5664. doi: 10.1021/ja069124n.

Calmettes, P., Durand, D., Desmadril, M., Minard, P., Receveur, V., and Smith, J.C. (1994). How random is a highly denatured protein? *Biophys Chem* 53(1-2)**,** 105-113. doi: 10.1016/0301-4622(94)00081-6.

Chi, Y.I., Frantz, J.D., Oh, B.C., Hansen, L., Dhe-Paganon, S., and Shoelson, S.E. (2002). Diabetes mutations delineate an atypical POU domain in HNF-1alpha. *Mol Cell* 10(5)**,** 1129-1137. doi: 10.1016/s1097-2765(02)00704-9.

Fitzkee, N.C., and Rose, G.D. (2004). Reassessing random-coil statistics in unfolded proteins. *Proc Natl Acad Sci U S A* 101(34)**,** 12497-12502. doi: 10.1073/pnas.0404236101.

Kind, L., Raasakka, A., Molnes, J., Aukrust, I., Bjorkhaug, L., Njolstad, P.R., et al. (2022). Structural and biophysical characterization of transcription factor HNF-1A as a tool to study MODY3 diabetes variants. *J Biol Chem* 298(4)**,** 101803. doi: 10.1016/j.jbc.2022.101803.

Manalastas-Cantos, K., Konarev, P.V., Hajizadeh, N.R., Kikhney, A.G., Petoukhov, M.V., Molodenskiy, D.S., et al. (2021). ATSAS 3.0: expanded functionality and new tools for small-angle scattering data analysis. *J Appl Crystallogr* 54(Pt 1)**,** 343-355. doi: 10.1107/S1600576720013412.

Piiadov, V., Ares de Araujo, E., Oliveira Neto, M., Craievich, A.F., and Polikarpov, I. (2019). SAXSMoW 2.0: Online calculator of the molecular weight of proteins in dilute solution from experimental SAXS data measured on a relative scale. *Protein Sci* 28(2)**,** 454-463. doi: 10.1002/pro.3528.

Raasakka, A., Linxweiler, H., Brophy, P.J., Sherman, D.L., and Kursula, P. (2019). Direct Binding of the Flexible C-Terminal Segment of Periaxin to beta4 Integrin Suggests a Molecular Basis for CMT4F. *Front Mol Neurosci* 12**,** 84. doi: 10.3389/fnmol.2019.00084.

Sanborn, A.L., Yeh, B.T., Feigerle, J.T., Hao, C.V., Townshend, R.J., Lieberman Aiden, E., et al. (2021). Simple biochemical features underlie transcriptional activation domain diversity and dynamic, fuzzy binding to Mediator. *Elife* 10. doi: 10.7554/eLife.68068.

Tria, G., Mertens, H.D., Kachala, M., and Svergun, D.I. (2015). Advanced ensemble modelling of flexible macromolecules using X-ray solution scattering. *IUCrJ* 2(Pt 2)**,** 207-217. doi: 10.1107/S205225251500202X.
